# CGRP at the Neuroimmune Interface: Regulator of Host Defense, Tumor Immunity, and Tissue Homeostasis

**DOI:** 10.34133/research.1270

**Published:** 2026-05-27

**Authors:** Cong Gao, Yue Shang, Xueyin Hu, Qianhui Wu, Longbo Ma, Bingwen Zou, Luntao Liu, Saijun Fan

**Affiliations:** ^1^ State Key Laboratory of Advanced Medical Materials and Devices, Tianjin Key Laboratory of Radiation Medicine and Molecular Nuclear Medicine, Tianjin Institutes of Health Science, Institute of Radiation Medicine, Chinese Academy of Medical Sciences & Peking Union Medical College, Tianjin 300192, China.; ^2^ Division of Thoracic Tumor Multimodality Treatment and Department of Radiation Oncology, Cancer Center, West China Hospital, Sichuan University, Chengdu 610041, China.

## Abstract

Sensory neurons act as crucial hubs for host defense, tumor immunity, and tissue homeostasis by detecting environmental cues and fine-tuning immune responses. Across a diversity of tissues, these neurons establish specialized neuroimmune units with resident immune cells, translating local stimuli into coordinated physiological adaptations. Central to this crosstalk is calcitonin gene-related peptide (CGRP). Released by activated sensory neurons, CGRP dynamically governs immune cell function, neurogenic inflammation, and tissue repair. This review synthesizes current evidence establishing CGRP as a master regulator of neuroimmune communication. We dissect its highly context-dependent roles in microbial infections, the tumor microenvironment, and barrier tissues, emphasizing how microbial identity, spatial niches, and concurrent signaling cues dictate its functional outcomes. Mechanistically, we explore the molecular cascades through which diverse cell types decode CGRP signals, focusing on receptor subtype selectivity and cell-specific adaptor switching. Furthermore, we evaluate emerging therapeutic strategies targeting the CGRP axis—ranging from small-molecule modulators to monoclonal antibodies—and their transformative potential for treating immune-mediated conditions, from chronic inflammation to malignancies. Ultimately, we identify critical knowledge gaps, such as the “CGRP receptor code” and neuromicrobial feedback loops, which must be resolved to fully harness the therapeutic promise of this neuroimmune axis.

## Introduction

As a critical bridge connecting the central nervous system (CNS) and peripheral tissues, the peripheral nervous system primarily orchestrates immunomodulation through localized neuroimmune interactions [1,2]. As the basic functional units of the peripheral nervous system, sensory neurons possess distinctive anatomical characteristics: Their somata reside within ganglia and exhibit a classic pseudounipolar morphology, with long peripheral processes specialized in peripheral signal reception and axons responsible for central transmission [3,4]. These neurons establish widely arborized peripheral processes that infiltrate cutaneous, visceral, and muscular tissues, enabling multimodal detection of mechanical, chemical, and thermal stimuli [5,6]. Signal propagation occurs via bidirectional antidromic axon reflexes, during which nerve terminals release a variety of neuropeptides and neurotransmitters that directly shape the peripheral immune microenvironment [6,7]. As specialized subpopulations of sensory neurons, nociceptors possess the ability to selectively detect potentially harmful stimuli when their intensity reaches the noxious range, subsequently transducing these stimuli into neural impulses [8–11]. Nociceptors are classically divided into 2 principal subtypes: medium-diameter myelinated Aδ fibers that mediate acute, well-localized fast pain, and small-diameter unmyelinated C-fibers responsible for diffuse, persistent slow pain [12,13]. Aδ nociceptors can be further subclassified into 2 distinct populations: Type I Aδ nociceptors exhibiting relatively high thermal thresholds (>50 °C) and Type II Aδ nociceptors with markedly lower thermal activation thresholds [11,14,15]. Unmyelinated C-fibers are similarly heterogeneous and can be categorized into functionally distinct subgroups based on their neuropeptide and receptor expression profiles [16]. The peptidergic C-nociceptor subpopulation is characterized by the release of 2 key neuropeptides, namely, substance P (SP) and calcitonin gene-related peptide (CGRP), together with the expression of tropomyosin receptor kinase A (TrkA) and trophic maintenance dependent on nerve growth factor (NGF) [17,18]. In contrast, nonpeptidergic nociceptors express the Rearranged during transfection receptor (Ret) and respond tothe glial cell line-derived neurotrophic factor [19–21].

**Fig. 1.**
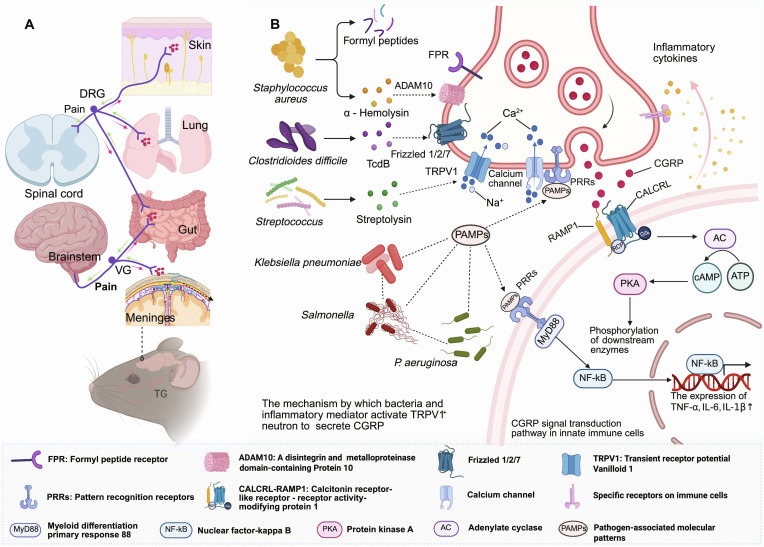
Bacteria activate sensory neurons that modulate immunity and inflammation. (A) Distribution of sensory neurons in organs such as the skin, lung, gut, and brain. (B) Mechanisms of sensory neuron activation during various bacterial infections and calcitonin gene-related peptide (CGRP)-mediated signal transduction in immune cells. This figure was created in BioRender. Gao, C. (2026) https://BioRender.com/8lutp8y (agreement number: ZY29I8ZNFY)

Nociceptors are widely distributed across diverse tissues, including the skin, mucosa, muscles, joints, and visceral organs [6]. The somata of these sensory neurons reside in distinct ganglia depending on their target innervation: dorsal root ganglia (DRG) for spinal projections, nodose/jugular vagal ganglia for brainstem connections, trigeminal ganglia for cranial structures, or within the myenteric and submucosal plexuses of the enteric nervous system [6,22] (Fig. 1A). The plasma membranes of nociceptors express a diverse array of receptors and ion channels that specifically respond to distinct noxious stimuli, including mechanical, chemical, and thermal stimuli [10]. Transient receptor potential vanilloid 1 (TRPV1), a member of the transient receptor potential ion channel family, functions as a nonselective cation channel permeable to sodium, calcium, and magnesium ions [23,24]. This structural property enables transmembrane flux of multiple ions, thereby modulating cellular membrane potential and function. TRPV1 is predominantly expressed in myelinated Aδ and unmyelinated C-sensory fibers of the DRG [25,26]. It serves as the receptor for capsaicin and a sensor for noxious heat (>43 °C). Under physiological conditions, it transduces harmful thermal stimuli into neural signals that propagate to the CNS, ultimately evoking pain perception and defensive behavioral responses [21,27]. Its responses to these stimuli are substantially regulated by the membrane potential; negative potential inhibits or prevents its response, while positive potential enhances its response [23,28,29]. Acid-sensing ion channels are proton-gated cation channels that function as extracellular pH sensors, activated under acidic conditions. Nav1.8, a voltage-gated sodium channel subtype, exhibits highly selective expression in the majority of C-nociceptors [21,30]. Upon activation of these channels, the influx of cations such as Na^+^ and Ca^2+^ induces membrane depolarization. When depolarization reaches threshold potential, action potentials propagate along nociceptor axons toward the CNS. At presynaptic terminals, these action potentials trigger vesicular release of neurotransmitters and neuropeptides, primarily including CGRP, SP, and glutamate [23]. This axonal reflex mechanism constitutes a critical molecular foundation for neuroimmune crosstalk.

CGRP, a 37-amino acid neuropeptide with pleiotropic physiological functions, is widely expressed across various human tissues and organ systems [31,32]. There are 2 evolutionarily conserved isoforms: α-CGRP and β-CGRP, encoded by distinct genetic loci (calcitonin-related polypeptide alpha [*Calca*] and calcitonin-related polypeptide beta [*Calca*], respectively) [33,34]. Despite sharing high sequence homology (>90% amino acid identity) and similar receptor binding affinities, these isoforms demonstrate remarkable tissue-specific expression patterns and exhibit nonredundant functional roles in vivo [32]. The α isoform is predominantly expressed in DRG and specific neuronal populations within the CNS, whereas the β isoform shows broader distribution across peripheral tissues, including thyroid C cells, epidermal keratinocytes, vascular endothelial cells, and immune cells, as well as the peripheral nervous system [32]. Notably, β-CGRP has been characterized as the “enteric form” of CGRP due to its predominant functional role in the enteric nervous system [23]. Similarly, CGRP receptors are widely distributed across multiple human organs and tissues [6]. Most importantly, the expression of CGRP receptors on various immune cells provides the fundamental physiological basis for CGRP-mediated neuroimmune crosstalk. To date, immune cell types reported to express CGRP receptors primarily encompass lymphocytes (group 2 innate lymphoid cells [ILC2s], CD4^+^T cells, CD8^+^T cells, and B cells) [35–37], macrophages [38,39], neutrophils [40], dendritic cells (DCs) [41], and monocytes [42]. CGRP exerts pleiotropic immunomodulatory functions through G protein-coupled receptor signaling, with distinct molecular mechanisms across different cell types. Specifically, CGRP binds to heterodimeric receptor complexes composed of calcitonin receptor-like receptor (CALCRL) and receptor activity-modifying protein 1 (RAMP1) on immune and nonimmune cells [32]. This interaction induces conformational changes in the G protein subunit, where RAMP1 determines ligand-binding specificity while CALCRL mediates G protein coupling and activation. Subsequent adenylate cyclase activation elevates intracellular cyclic adenosine monophosphate (cAMP) levels, leading to protein kinase A (PKA) activation. PKA then phosphorylates multiple downstream effector proteins, thereby regulating gene transcription and inflammatory responses across physiological systems [6,10].

Accumulating evidence has highlighted the multifaceted roles of CGRP-mediated neuroimmune homeostasis axis (CGRP–NIHS Axis), yet several critical scientific contradictions and unresolved questions remain, which constitute the core focus of this review. First, CGRP exhibits contradictory roles in different pathological contexts—while it may facilitate pathogen clearance during infection, it often exerts immunosuppressive effects in tumor microenvironments (TMEs), and the underlying mechanisms driving this functional divergence remain unclear. Second, the bidirectional immunomodulatory capacity of CGRP (i.e., promoting or inhibiting inflammatory responses) displays striking tissue specificity, but how tissue microenvironment cues shape this regulatory bias and whether distinct CGRP isoforms (α/β) are involved in this process require systematic clarification. Third, the molecular crosstalk between CGRP signaling and key immune cell subsets (e.g., macrophages and T cells) in maintaining barrier homeostasis (e.g.,the skin and the intestinal mucosa) is not fully elucidated, especially regarding the interplay with other neuropeptides and ion channels (e.g., TRPV1). Against this backdrop, this review systematically analyzes the multidimensional roles of the CGRP–NIHS Axis. First, we elucidate the sophisticated mechanisms by which the CGRP–NIHS Axis orchestrates local immune microenvironments. Second, we synthesize recent discoveries revealing the dual immunomodulatory capacity of the CGRP–NIHS Axis in pathogen clearance, tumor immunity, and barrier homeostasis. Finally, we propose therapeutic strategies targeting the CGRP–NIHS Axis, providing novel insights for the treatment of infectious diseases, autoimmune disorders, and malignancies. Investigating this paradigm of peripheral neuro-immune crosstalk not only expands the theoretical framework of classical neuroimmunology but also establishes a scientific foundation for developing tissue-specific immunomodulatory therapies.

## Regulatory Role of CGRP in Host Defense During Infectious Diseases

### Spatiotemporal dynamics of the neuroimmune interface: CGRP as a microenvironmental rheostat in bacterial infections

In barrier tissues such as the skin, respiratory tract, gastrointestinal tract, and urinary tract, which are perpetually exposed to bacterial pathogens, densely distributed sensory neurons are intricately intertwined with resident immune cells, forming highly integrated neuroimmune cell units [43–45]. Traditional immunological views posited that infection-induced pain was merely a secondary byproduct of immune activation. However, emerging evidence suggests that bacterial pathogens can directly stimulate nociceptors and trigger a massive release of neuropeptides, acting entirely independently of the host immune system [46,47]. The pathways by which pathogens activate sensory neurons exhibit high microbial specificity and complex molecular mechanisms. Gram-positive bacteria primarily activate neurons through secreted exotoxins and metabolites [48,49]. For instance, *Staphylococcus aureus* activates TRPV1^+^ neurons via 2 distinct mechanisms: Its secreted N-formylated peptides bind directly to formyl peptide receptors on neurons [50–52], while the pore-forming toxin α-hemolysin creates membrane pores through interaction with a disintegrin and metalloproteinase domain-containing protein 10 (ADAM10), leading to a massive influx of CA^2+^ [53,54]. Similarly, *Clostridioides difficile* TcdB directly stimulates intestinal sensory neurons by binding to Frizzled 1/2/7 (FZD1/2/7) receptors [55–57], and Streptolysin S activates TRPV1^+^ neurons through cholesterol-dependent membrane perforation [58]. Conversely, gram-negative bacteria activate sensory neurons primarily through pathogen-associated molecular patterns such as lipopolysaccharide (LPS) and flagellin. Furthermore, these bacteria can activate the myeloid differentiation primary response 88-dependent nuclear factor kappa B (NF-κB) pathway in immune cells, inducing the production of numerous inflammatory cytokines that indirectly sensitize sensory neurons via paracrine signaling [42]. Ultimately, whether through direct provocation or indirect sensitization, these interactions culminate in the synergistic release of neuropeptides, including CGRP, SP, and vasoactive intestinal peptide (VIP) (Fig. 1B). In this complex cotransmission network, CGRP serves as the ultimate “microenvironmental rheostat”, leveraging its ability to profoundly reprogram various leukocyte subpopulations to modulate infection progression and tissue homeostasis.

Superficially, the roles of CGRP—alternating between suppressing defense and driving inflammation across different infection scenarios—appear paradoxical. However, this “contradiction” stems fundamentally from its precise coordination of the spatiotemporal dynamics within the infection niche. This rheostat function is not static; it strictly follows the kinetic trajectory of innate immunity—from the rapid response of early “pioneer” cells to the macrophage-dominated resolution of the intermediate phase of inflammation, and finally to the immune evasion of high-virulence pathogens in late stages. Essentially, this mirrors a protracted struggle for “neuromodulatory control” between the host defense system and invading pathogens.

**Fig. 2.**
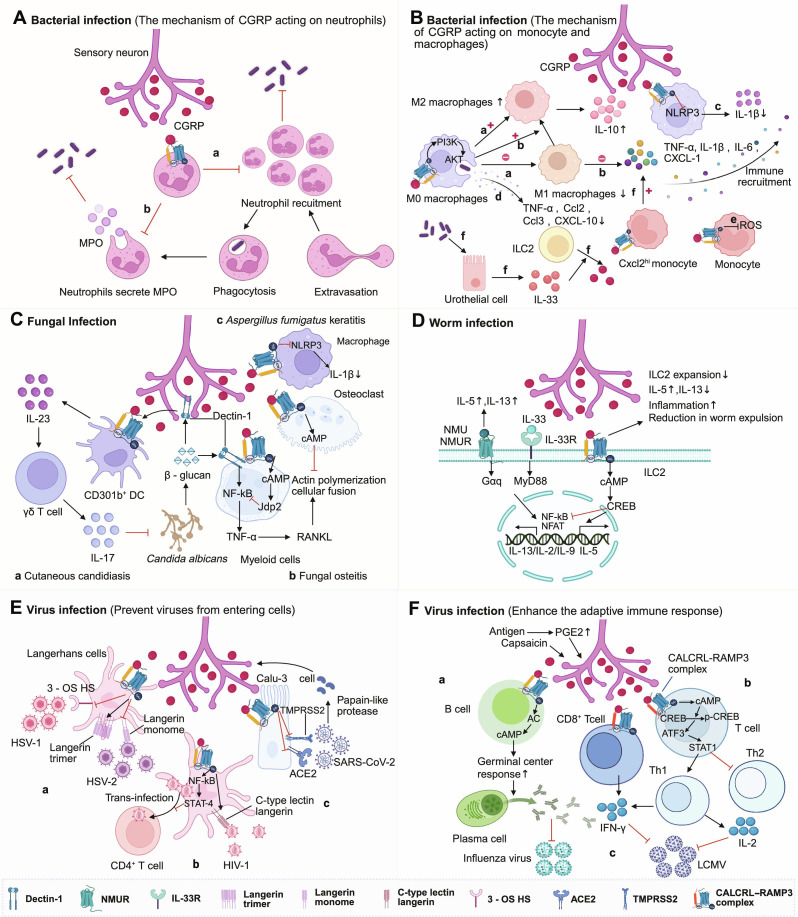
Mechanisms of calcitonin gene-related peptide (CGRP)-immune cell interactions in infectious diseases. (A) The mechanism of CGRP acting on neutrophils. a. CGRP suppresses neutrophil recruitment during bacterial skin/lung infections (neutrophil recruitment↓); b. CGRP inhibits neutrophil infiltration and myeloperoxidase (MPO) secretion (MPO↓); (B) The mechanism of CGRP acting on monocyte and macrophages. a. CGRP skews M0 macrophage polarization from proinflammatory M1 to anti-inflammatory M2 phenotypes (M1↓, M2↑), dampening inflammatory responses. b. CGRP-calcitonin receptor-like receptor (CALCRL)/receptor activity-modifying protein 1 (RAMP1) activates phosphoinositide 3-kinase (PI3K)-AKT signaling to drive M1-to-M2 transition. c. *Klebsiella pneumoniae* pneumonia: CGRP inhibits NLR family pyrin domain containing 3 (NLRP3) inflammasome activation and interleukin-1β (IL-1β) maturation (IL-1β↓). d. CGRP suppresses proinflammatory cytokine/chemokine secretion (proinflammatory cytokine/chemokine↓) in meningeal macrophages (bacterial meningitis) and bone marrow-derived macrophages (BMDMs, carbapenem-resistant *Klebsiella* pneumoniae [CRKP] lung infection). e. CRKP pulmonary infection: CGRP impairs monocyte ROS production via RAMP1 (ROS↓). f. In bacterial cystitis, urothelial IL-33-ST2 signaling induces group 2 innate lymphoid cell (ILC2)-derived CGRP, which stimulates Cxcl2^hi^ monocytes to exacerbate inflammation. (C) Antifungal immune regulation: a. Cutaneous candidiasis: *Candida albicans* β-glucan activates nociceptors via Dectin-1-PLC-transient receptor potential vanilloid 1 (TRPV1)/transient receptor potential ankyrin 1 (TRPA1) axis to trigger CGRP release. CGRP drives CD301b^+^ dendritic cells (DCs) to produce IL-23 (IL-23↑), promoting dermal γδ T cell-derived IL-17 production (IL-17↑). b. Fungal osteomyelitis: CGRP up-regulates Jun dimerization protein 2 (Jdp2) (Jdp2↑) to suppress Dectin-1-mediated inflammation and receptor activator of nuclear factor kappa-B ligand (RANKL)-induced osteoclast multinucleation (osteoclast multinucleation↓). c. *Aspergillus fumigatus* keratitis: CGRP down-regulates macrophage NLRP3 expression (NLRP3↓), attenuating inflammation. d. Helminth infection: CGRP negatively regulates ILC2 responses (ILC2 responses↓) in gut/lung, impairing worm expulsion. (D) CGRP exerts its effects via the cyclic adenosine monophosphate (cAMP)-cAMP response element-binding protein (CREB) signaling pathway: It synergizes with IL-33 to promote IL-5 expression (IL-5↑), while antagonizing the effects of neuromedin U (NMU) and IL-33 to inhibit IL-13 production (IL-13↓) and ILC2 proliferation (ILC2 proliferation↓). (E) a. CGRP protects human Langerhans cells from infection by blocking herpes simplex virus type 1 (HSV-1) and herpes simplex virus type 2 (HSV-2) entry through down-regulating 3-O-sulfated heparan sulfate (3-OS HS) (3-OS HS↓) and up-regulating the expression of atypical langerin double trimers (atypical langerin double trimers↑), respectively. b. CGRP suppresses HIV-1 trans-infection of CD4^+^ T cells from Langerhans cells via the NF-κB/signal transducer and activator of transcription 4 (STAT4) signaling pathway; c. Furthermore, CGRP reduces the expression of SARS-CoV-2 entry receptors angiotensin-converting enzyme 2 (ACE2) (ACE2↓) and transmembrane protease serine 2 (TMPRSS2) (TMPRSS2↓) on Calu-3 cells, thereby blocking viral entry (viral entry↓) and inhibiting infection. (F) CGRP acts on CALCRL–RAMP1 receptors on B cells to promote antiviral humoral immune responses. It also binds to CALCRL–RAMP3 receptors on T cells and CD8^+^ T cells, promoting the differentiation of T cells into type 1 helper T (Th1) cells (Th1 cells↑) and enhancing the cytotoxic responses of CD8^+^ T cells (CD8^+^ T cell cytotoxicity↑). This figure was created in BioRender. Gao, C. (2026) https://BioRender.com/a29e118 (agreement number: FR29I8ZMH5)

In the hyperacute phase of this struggle, the host faces a dilemma: rapid pathogen clearance versus the prevention of catastrophic tissue damage. Here, the neuropeptide network exhibits precise spatiotemporal division of labor. SP typically acts as an immunological “accelerator”, responding first by binding to high-affinity neurokinin-1 receptors (NK-1Rs). This not only stimulates natural killer (NK) cells to produce interferon-γ (IFN-γ) for rapid antimicrobial defense [59] but also extensively activates NF-κB pathways involved in local stromal remodeling [60]. However, the intense inflammatory storm driven by SP can trigger maladaptive responses, such as osteoclast-mediated bone destruction during *S. aureus* infection [61], or even be hijacked by pathogens to enhance their virulence and tissue adherence [62]. To counterbalance this destructive pioneer signal, CGRP follows as an endogenous “counter-regulatory brake”, primarily limiting neutrophil function to prevent extensive tissue necrosis [63]. In *S. aureus*-induced skin or lung infections, CGRP secreted by TRPV1^+^ neurons markedly inhibits neutrophil recruitment to the infection site [48,63] (Fig. 2A, a); in cutaneous *Streptococcus* infections, CGRP further down-regulates the expression of myeloperoxidase, a key antimicrobial enzyme secreted by neutrophils (Fig. 2A, b). While this braking mechanism objectively slows early bacterial clearance, its core evolutionary significance lies in protecting the host from lethal immunopathological damage [58]. When this “brake” is removed—via genetic ablation of TRPV1^+^ neurons or the use of CGRP release inhibitors like botulinum neurotoxin A (BoNT/A) and receptor antagonists like BIBN4096 (olcegepant)—neutrophil recruitment surges, leading to a considerable reduction in bacterial load within the infected tissue [64].

As infection enters the mid-term phase, the peptide network’s regulation of innate immunity transcends simple recruitment limits, exerting a profound orchestration of tissue homeostasis. At the turning point between bacterial clearance and tissue repair, various neuropeptides synergistically maintain stability across different anatomical layers. For example, during *Citrobacter rodentium* intestinal infection, VIP protects epithelial mitochondria from damage induced by IFN-γ and tumor necrosis factor-α (TNF-α) [65]; in bacterial keratitis, VIP stimulates the secretion of growth factors like epidermal growth factor and fibroblast growth factor to prevent corneal perforation [66]. As the core of this stabilizing network, CGRP potently blocks the polarization of uncommitted macrophages (M0) toward the proinflammatory M1 phenotype, while driving their polarization toward the anti-inflammatory and reparative M2 phenotype. In mouse models lacking TRPV1 signaling, local M1 macrophage infiltration increases markedly, causing a surge in proinflammatory interleukin-1β (IL-1β) and TNF-α while IL-10 levels plummet [48] (Fig. 2B, a). This polarization remodeling is decisive in specific mucosal tissues; for instance, in *Pseudomonas aeruginosa* keratitis, CGRP released from the trigeminal ganglion relies on the CALCRL–RAMP1 complex on macrophages to activate the phosphoinositide 3-kinase-protein kinase B (AKT) signaling pathway, driving M2 conversion [67] (Fig. 2B, b), which, alongside mediators like VIP, rescues visual function [66]. Remarkably, CGRP also acts as an “intracellular checkpoint” by directly intervening in inflammasome assembly to achieve deep stabilization. In the *Klebsiella pneumoniae* lung infection microenvironment, CGRP endocytosed by macrophages penetrates the cytosol and binds with high affinity to the NLR family pyrin domain containing 3 (NLRP3) sensor. Since the mitotic kinase NIMA-related kinase 7 is an indispensable prerequisite for NLRP3 oligomerization [68], the physical binding of CGRP competitively displaces NIMA-related kinase 7, effectively dismantling the foundation for the assembly of the inflammasome and blocking the release of mature IL-1β [68], which is crucial for leukocyte recruitment [69,70] (Fig. 2B, c). Experimental evidence confirming that CGRP antagonists lacking the first 7 amino acids (CGRP[8-37]) can considerably improve infection control in mice provides definitive proof that CGRP’s immunosuppressive effects are rooted in this intracellular structural intervention [68].

However, as infections evolve into late stages, this neural dominance intended for homeostasis is often opportunistically hijacked by high-virulence pathogens to facilitate immune evasion. Just as the mucosal regulatory pathways of VIP are hijacked by *Bordetella* via its type III secretion system to promote long-term colonization [71], CGRP’s potent immunosuppressive functions face similar passive exploitation. During *Streptococcus* pneumoniae or Group B *Streptococcus* infections, pathogens pathologically activate the trigeminal ganglion to release CGRP, which acts on meningeal macrophages to systematically shut down the secretion of critical chemokines such as TNF-α, CCL2, CCL3, and CXCL10. This pathogen-induced chemostatic inhibition creates an “immune-privileged” niche, greatly facilitating bacterial penetration of the meningeal barrier and leading to fatal infections [72] (Fig. 2B, d). Similarly, in carbapenem-resistant *Klebsiella pneumoniae* infections, host Ly6G^+^ monocytes are induced to up-regulate Calcrl expression, allowing high concentrations of airway CGRP to profoundly suppress reactive oxygen species production and the direct bactericidal capacity of these cells, thereby accelerating the progression of sepsis [42] (Fig. 2B, e).

Breaking the traditional stereotype of CGRP as solely immunosuppressive, its performance in specific microenvironments further highlights the complexity of this struggle for neural dominance. In models of bacterial cystitis, the source and function of neuropeptides undergo a subversive reversal: IL-33 secreted by the urothelium under bacterial stimulation activates ST2 receptors on ILC2s, triggering a massive release of non-neuronal CGRP. These CGRP molecules paradoxically act as “proinflammatory accelerators”, acting on local Cxcl2^high^ monocytes to drive an explosion of proinflammatory cytokines like IL-1β and IL-6. Targeted depletion of monocytes using clodronate liposomes (Clod) prior to infection markedly alleviates bladder inflammation and voiding dysfunction, with the proinflammatory effects of exogenous CGRP completely vanishing. Crucially, this process does not affect the final bacterial colony-forming unit load [37] (Fig. 2B, f). This mechanistic separation of “pain perception” and “pathogen clearance” holds profound clinical translational value. Drastic fluctuations at the neuroimmune interface are not limited to the local niche but serve as systemic biomarkers in patients. Clinical studies indicate that in children with community-acquired pneumonia, plasma levels of neurogenic inflammatory peptides like CGRP, SP, and VIP undergo substantial changes, showing potential diagnostic value in distinguishing viral from bacterial infections [73]. This further confirms that in actual human infections, neuropeptides dictate the evolution of inflammatory pathology and systemic symptoms. Consequently, the “decoupling” of pathological symptoms from pathogen clearance via pharmacological means becomes viable. Mechanistically, this decoupling benefits from the differential responses of various immune cells to neuropeptides within the microenvironment. While blocking CGRP signaling successfully “silences” the inflammatory storm and pain sensitization driven by monocytes/macrophages, it simultaneously releases the “brake” on early neutrophil recruitment, allowing pioneer phagocytes to clear pathogens unhindered. Based on this, the use of highly selective CGRP receptor antagonists (e.g., BIBN4096) can effectively improve inflammation-related complications and pain without compromising the host’s innate antimicrobial defenses, providing a robust theoretical foundation for reevaluating and broadening the indications for CGRP-targeted drugs in infectious diseases [74].

### Early warning and mobilization in fungal infections: The neuropeptide-driven axis of type 17 protective immunity and tissue homeostasis

In the microenvironment of fungal infections (e.g., *Candida albicans* and *Aspergillus fumigatus*), neuropeptides exhibit a regulatory logic distinct from that observed in bacterial challenges: They function not merely as anti-inflammatory “brakes” but more prominently as “early-warning systems” that drive protective host immunity. Research has revealed that core components of the fungal cell wall, such as β-glucans, can directly activate TRPV1^+^/transient receptor potential ankyrin 1 (TRPA1)^+^ nociceptors in the skin, lungs, and bone tissues. This stimulation, mediated through the Dectin-1-transient receptor potential pathway, typically precedes traditional innate immune recognition, thereby providing critical early-warning signals for barrier tissues [41,75,76]. Concurrently, these neurogenic signals play a refined role in maintaining the homeostasis of commensal mycobiota. For instance, the CGRP–RAMP1 axis precisely tunes cutaneous adaptive immunity to commensals, ensuring that the host limits fungal overgrowth while precluding pathological autoimmune damage [77].

The CGRP released from sensory nerve endings serves as the primary trigger for initiating type 17 protective immunity, a regulatory axis that exhibits high conservation and universality across respiratory barriers such as the lungs. Specifically, CGRP acts directly on RAMP1 receptors on the surface of CD301b^+^ dermal DCs or lung-resident immune cells. By elevating intracellular cAMP levels, it potently drives these cells to secrete the cytokine IL-23. Subsequently, IL-23 activates local γδ T cells or innate lymphoid cells (ILCs), inducing them to produce substantial quantities of IL-17A. This cascade considerably enhances neutrophil recruitment and antimicrobial peptide production, establishing a robust defense line against fungal invasion [41,76] (Fig. 2C, a). Remarkably, this protective mechanism possesses a unique “anticipatory” quality: Through antidromic action potential conduction in neurons, neural activation at an infected site can trigger CGRP release in adjacent, uninfected regions. This mechanism primes neighboring tissues before the actual dissemination of pathogens, forming a regional “anticipatory immunity” that effectively halts fungal spread across tissues [76].

The homeostatic functions of neuropeptides exhibit exquisite tissue specificity and molecular targeting across different anatomical compartments. In models of *Candida*-induced fungal osteomyelitis, CGRP acts on G-protein-coupled receptors to elevate cAMP levels in bone marrow cells, subsequently inducing the expression of the transcriptional repressor Jun dimerization protein 2. Jun dimerization protein 2 binds to the NF-κB p65 subunit, inhibiting its association with proinflammatory cytokine promoters and thus reducing Dectin-1-mediated proinflammatory cytokine production. Furthermore, CGRP interferes with the multinucleation of osteoclasts duringRANKL (receptor activator of nuclear factor kappa-B ligand)-induced osteoclastogenesis by inducing cAMP to inhibit actin ring formation, ultimately facilitating the resolution of fungal osteomyelitis [75] (Fig. 2C, b). In *A. fumigatus*-induced keratitis, CGRP and VIP collaboratively exert pivotal anti-inflammatory effects. CGRP modulates inflammasome assembly by inhibiting the activation of the NLRP3–Caspase-1–IL-1β signaling pathway, subsequently down-regulating various proinflammatory cytokines and chemokines [78,79] (Fig. 2C, c). Concurrently, exogenous VIP treatment has been shown to considerably reduce proinflammatory cytokine levels in the infected cornea and attenuate tissue edema... thereby preserving its transparency and visual function [80].

Notably, neuropeptides maintain a delicate equilibrium between systemic inflammation and antifungal immunity. For instance, VIP demonstrates considerable anti-inflammatory and antiarthritic effects in collagen-induced arthritis, primarily by suppressing the production of proinflammatory cytokines such as TNF-α. Although this immunosuppression could theoretically compromise host defense, VIP treatment in *C. albicans* infection models did not substantially impair the host’s ability to clear renal fungal colonization. This indicates that VIP can attenuate autoimmune pathological damage while retaining essential antifungal defense efficacy [81].

In summary, CGRP and VIP facilitate a rapid transition from nociception to protective immunity by precisely remodeling the function of neuroimmune cell units during fungal challenge. This seamless integration of “early warning”, “effector mobilization”, and “deep stabilization” not only elucidates the evolutionary significance of the intense pain associated with fungal infections but also provides a novel theoretical dimension for pharmacologically modulating neuropeptide signaling to bolster antifungal immunity while suppressing immunopathology.

### Neuroimmune equilibrium in parasitic infections: From feedback inhibition to homeostatic protection

In the intricate landscape of parasitic infections—particularly intestinal helminthiasis and cutaneous leishmaniasis—neuroimmune crosstalk manifests as a sophisticated “braking” logic, which stands in stark contrast to the neuropeptide-driven acceleration of type 17 immunity observed in fungal challenges [82,83]. The host defense against helminths, such as *Nippostrongylus brasiliensis*, predominantly relies on type 2 immune responses. Recent breakthroughs have elucidated a pivotal negative feedback loop: The helminth-induced surge in IL-13 production from ILC2s is directly perceived by IL-13 receptors expressed on intrinsic enteric neurons. In response, these neurons markedly up-regulate Calcb expression and release β-CGRP, which targets RAMP1 receptors on ILC2s. This signaling axis elevates intracellular cAMP levels and partially activates the cAMP response element-binding protein, thereby suppressing ILC2 proliferation and the secretion of effector cytokines such as IL-13 [82] (Fig. 2D).

Within this regulatory network, CGRP functions as a “homeostatic rheostat” that operates through a molecular tug-of-war with neuromedin U. While neuromedin U synergizes with IL-33 to amplify type 2 responses via Gαq-mediated nuclear factor of activated T cells (NFAT) activation, CGRP serves as a critical counter-regulator that constrains this proinflammatory program. Although this CGRP-driven inhibition may theoretically impair the immediate rate of pathogen clearance, its core evolutionary significance lies in preventing irreversible tissue damage caused by exaggerated type 2 responses [82–85]. Drawing parallels from broader enteric mucosal models, the nervous system achieves higher-order strategic orchestration: Enteric sensory neurons can sense cytokine cues and modulate the release of neuropeptides, including VIP, to enhance the mucosal barrier’s “anticipatory immunity” by regulating group 3 innate lymphoid cell (ILC3) recruitment [86]. This intricate interplay ensures a delicate survival equilibrium between effective pathogen elimination and the preservation of tissue integrity [87]. The universality of such neurogenic modulation is further evidenced in cutaneous parasitic infections, such as leishmaniasis, where distinct neuropeptides exert divergent effects on disease progression. External stressors can profoundly alter the local neuroimmune microenvironment via the neural axis: The up-regulation of SP is closely associated with the aberrant migration of Langerhans cells (LCs) and clinical exacerbation of lesions. In contrast, CGRP predominantly fine-tunes the intensity of local inflammatory infiltration [88]. Collectively, these findings suggest that at the anatomical outposts of parasitic invasion, neuropeptides construct a “sensing-feedback” axis that constrains immune responses within safe homeostatic thresholds. This seamless transition from inflammatory sensing to neuropeptide-mediated feedback not only reflects the evolutionary wisdom of host adaptation to chronic infection but also provides a compelling blueprint for pharmacological intervention in type 2 inflammatory disorders [82,88].

### Neuroimmune orchestration in viral infections: From molecular entry blockade to systemic adaptive immunity

In the complex landscape of viral defense, CGRP, SP, and VIP constitute a sophisticated neuropeptide regulatory matrix. At the anatomical outposts of viral invasion, CGRP serves as a molecular gatekeeper by exerting highly subtype-specific protective effects on human LCs; this protection is particularly pronounced in monocyte-derived LCs and langerin^high^ epidermal LCs, while showing no marked effect on langerin^low^ populations [89]. For *herpes simplex virus type 1* (*HSV-1*), which typically utilizes a pH-dependent entry mechanism and specific receptors for 3-O-sulfated heparan sulfate, CGRP considerably suppresses infection by down-regulating 3-O-sulfated heparan sulfate expression and abrogating the pH dependency of the fusion process [89–92]. Conversely, for *herpes simplex virus type 2* (*HSV-2*), which enters via pH-independent, langerin receptor-mediated endocytosis, CGRP does not alter total receptor levels but instead induces the cell-surface expression of conformationally atypical langerin double trimers. These defective structures exhibit impaired ligand-binding affinity, thereby physically shielding LCs from *HSV-2* attachment and subsequent entry [89,93,94] (Fig. 2E, a). This defensive paradigm extends to *human immunodeficiency virus type 1* (*HIV-1*) transmission, where native CGRP and its stable analog SAX up-regulate langerin expression to facilitate the internalization and lysosomal degradation of virions. Furthermore, CGRP activates a noncanonical NF-κB/signal transducer and activator of transcription 4 signaling pathway, reducing the expression of adhesion molecules and preventing the formation of LC-CD4^+^ T cell conjugates, thus curbing the lateral trans-infection of *HIV-1* from the mucosa to systemic circulation [95] (Fig. 2E, b).

Simultaneously, SP acts in synergy with CGRP during the early stages of infection to augment the production of proinflammatory cytokines, such as TNF-α and IL-1β, in macrophages, thereby assisting the host in establishing an immediate antiviral state [96]. However, this neurogenic inflammatory mobilization can be a double-edged sword; in chronic CNS infections, such as those caused by *Borna disease virus* (*BDV*), the sustained up-regulation of SP and CGRP is closely associated with the exacerbation of pathological neuroinflammation [97]. Within the respiratory tract, this regulatory axis manifests as a higher-order homeostatic control: Viral proteases can directly trigger CGRP secretion from lung-innervating sensory neurons, which in turn down-regulates angiotensin-converting enzyme 2 and transmembrane protease serine 2 expression to establish a neuro-derived barrier against severe acute respiratory syndrome coronavirus 2 (*SARS-CoV-2*) entry [98] (Fig. 2E, c). Complementing this, clinical evidence reveals that VIP functions as a critical endogenous protective factor, with its elevated levels strongly correlating with increased survival and reduced viral-induced damage in severe COVID-19 patients [99].

In the realm of systemic immunity, CGRP profoundly reshapes the adaptive immune landscape. CGRP derived from splenic nociceptors targets B cells via the CALCRL–RAMP1 receptor complex, activating the cAMP pathway to markedly promote germinal center reactions and robust humoral responses against influenza viruses [100] (Fig. 2F, a). Regarding cellular immunity, CGRP and its downstream cAMP response element-binding protein-activating transcription factor 3-signal transducer and activator of transcription 1 signaling axis precisely orchestrate T cell differentiation. This molecular cascade biases T cell commitment toward type 1 helper T (Th1) and cytotoxic CD8^+^ T (Tc1) phenotypes—characterized by elevated IFN-γ production—while antagonizing type 2 helper T (Th2) differentiation, thus accelerating the clearance of viruses such as *lymphocytic choriomeningitis virus* (*LCMV*) (Fig. 2F, b and c). Experimental evidence from *Calca*^−^/^−^ or *Ramp3*^−^/^−^ mice further underscores this necessity, as these deficiencies result in impaired antiviral functional profiles, splenomegaly, and delayed viral clearance [101]. Collectively, this regulatory network—coordinated by CGRP and assisted by the divergent roles of SP and VIP—ensures that the host achieves a dynamic equilibrium between effective pathogen elimination and the preservation of tissue homeostasis across diverse viral challenges.

## Calcitonin Gene-Related Peptide Participates in Regulating Barrier Tissue Homeostasis and Repair

### CGRP maintains intestinal epithelial barrier homeostasis and regulates intestinal microbiota

As the largest mucosal immune organ and central metabolic hub in the human body, the gastrointestinal tract is continuously exposed to a complex milieu comprising dietary components, commensal microbiota, and opportunistic pathogens [102]. The intricate synergy between the nervous and immune systems enables continuous surveillance and adaptive regulation of luminal contents, a process indispensable for the maintenance of intestinal barrier defense [103,104]. The gut is dually innervated by extrinsic innervation (peripheral sensory and autonomic nerves) and intrinsic innervation (the enteric nervous system). The dynamic crosstalk among these neural projections, intestinal epithelial cells, immune populations, and the gut microbiota collectively dictates physiological homeostasis and the integrity of antimicrobial defense barriers [105–107].

Within this regulatory matrix, sensory neurons emerge as critical architects in preserving tissue barrier integrity and modulating intestinal inflammation. Extrinsic sensory neurons innervating the gut reside in the DRG and vagal ganglia, conveying viscerosensory signals to the spinal cord and brainstem, respectively [6,108]. Conversely, intrinsic sensory neurons are embedded within the myenteric and submucosal plexuses [23]. In murine models, vagal sensory afferents predominantly innervate the proximal colon, whereas DRG-derived sensory neurons primarily supply the jejunum and the distal colon [109,110]. Both intrinsic and extrinsic enteric sensory neurons express pattern recognition receptors, empowering them to directly sense microbial cues and subsequently trigger the release of neuropeptides, notably CGRP [111,112]. In this context, CGRP transcends the role of a unidirectional inflammatory mediator; instead, it operates as a microenvironment-calibrated “immune orchestrator”. Through precise interactions with the intestinal epithelium, mucosal immune cells, and commensal microbiota, CGRP exerts highly context-dependent, dual regulatory functions. Consequently, it profoundly shapes the trajectory and ultimate resolution of intestinal infections, inflammatory responses, and microbial dysbiosis [113].

**Fig. 3.**
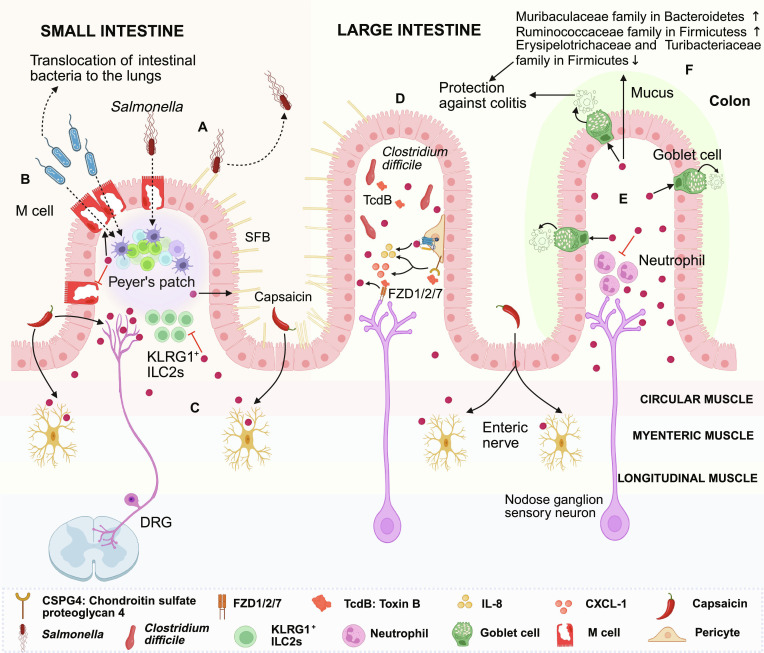
Calcitonin gene-related peptide (CGRP) is involved in maintaining intestinal barrier homeostasis. (A) In the ileum of the small intestine, dorsal root ganglia (DRG) transient receptor potential vanilloid 1 (TRPV1)^+^ Nav1.8^+^ neurons can defend against Salmonella invasion by releasing CGRP to limit the number of M cells (Salmonella invasion sites, M cells↓) and increase the number of segmented filamentous bacteria (SFB) (SFB↑). (B) CGRP secreted after nociceptor activation reduces the migration of intestinal bacteria to the lungs (bacteria migration↓) by decreasing the number of M cells (M cells↓) and restoring the antigen-presenting function of M cells. (C) In an ovalbumin (OVA)-induced mouse food allergy model, ChAT^+^ enteric neurons secrete CGRP, which directly acts on specific receptors on group 2 innate lymphoid cells (ILC2s) to inhibit the proliferation and activation of killer cell lectin-like receptor G1 (KLRG1)^+^ ILC2s (KLRG1^+^ ILC2 proliferation↓) and the expression of interleukin-5 (IL-5) (IL-5↓). (D) TcdB triggers neurogenic inflammation by targeting intestinal sensory neurons and pericytes. Additionally, CGRP can further promote IL-8 release (IL-8↑) by activating pericytes via calcitonin receptor-like receptor (CALCRL)-receptor activity-modifying protein 1 (RAMP1) receptors. (E) In the colon, nociceptive neurons mediate colitis protection by regulating mucus secretion from goblet cells and the intestinal microbiota through the CGRP–RAMP1 axis. (F) The release of CGRP alters the composition of the intestinal microbiota. This figure was created in BioRender. Gao, C. (2026) https://BioRender.com/n1mydw0 (agreement number: NB29I8ZLSI)

In response to intestinal pathogenic challenges, distinct neuropeptides orchestrate divergent yet complementary defensive strategies. For instance, VIP interacts with ILC3s to promote the production of interleukin-22 (IL-22), thereby endowing the gut with “anticipatory mucosal immunity” that fortifies the barrier even before pathogen (e.g., *Citrobacter rodentium*) invasion [114,115]. Conversely, during bacterial infection, SP typically drives robust acute proinflammatory responses and immune cell recruitment; however, its excessive release can culminate in maladaptive immunopathological damage to the host [116]. Distinct from VIP’s homeostatic maintenance and SP’s intense proinflammatory drive, CGRP exhibits a highly precise, targeted anti-infective efficacy. In the ileum, DRG neurons expressing TRPV1 and voltage-gated sodium channel 1.8 (Nav1.8) mediate host defense against *Salmonella* through CGRP release [117]. *Salmonella* exploits its type 1 fimbrial adhesin FimH to bind glycoprotein 2 on the surface of microfold (M) cells, facilitating ileal invasion. Concurrently, segmented filamentous bacteria (SFB), which colonize the ileal villi and the epithelium associated with Peyer’s patches, can suppress pathogenic colonization by competing for nutrients and secreting metabolites or toxins that modulate host antimicrobial responses. Consequently, host defense mechanisms against *Salmonella* primarily involve 2 avenues: reducing the availability of M cell invasion sites or increasing the abundance of SFB. Enteric *Salmonella* infection activates TRPV1^+^ nociceptors within the DRG; the subsequent release of CGRP diminishes the number of M cells while elevating SFB levels, thereby restricting *Salmonella* infection [117,118] (Fig. 3A). This CGRP-driven barrier protection extends beyond localized effects to exert cross-organ, systemic significance. In models of severe traumatic brain injury, impaired peripheral nociceptive signaling leads to an aberrant expansion of M cells coupled with compromised antigen-presentation function. This defect triggers the gut-to-lung translocation of enteric pathogens, culminating in lethal pulmonary infections. Inducing CGRP secretion effectively curtails this abnormal M cell proliferation, restores their major histocompatibility complex class II expression, and elevates local secretory immunoglobulin A (IgA) (sIgA) levels. These findings underscore CGRP’s critical role as a regulatory “valve” in obstructing the migratory routes of enteric pathogens to the lungs [119] (Fig. 3B).

In the maintenance of intestinal immune homeostasis, the functional trajectory of the neuropeptide network is profoundly dictated by the specific pathological microenvironment. In scenarios of excessive type 2 inflammation, such as food allergy, enteric neuron-derived α-CGRP precisely modulates type 2 immunity—analogous to the barrier-protective logic of VIP-activated ILC3s. Here, CGRP acts as a potent “immunosuppressant”, directly targeting ILC2s. By activating the cAMP signaling pathway, it substantially curtails the proliferation and activation of killer cell lectin-like receptor G1 (KLRG1)^+^ ILC2s, thereby preventing excessive mucosal immunopathology [120] (Fig. 3C). Nevertheless, CGRP is not an absolute protective factor; under specific pathogenic contexts, it can synergize with SP to become a “proinflammatory” driver. For example, during dysbiosis induced by *Clostridioides difficile* infection, the bacterial toxin B (TcdB) targets both enteric sensory neurons and pericytes, eliciting neurogenic inflammation. On one hand, the toxin binds to FZD1/2/7 receptors on sensory neurons, simultaneously stimulating the release of both SP and CGRP, with SP rapidly initiating an acute inflammatory cascade. On the other hand, CGRP uniquely activates pericytes via the CALCRL–RAMP1 complex, further promoting the secretion of the chemokine CXCL1 and IL-8, thereby amplifying the inflammatory response. Blocking either the SP or CGRP signaling pathways mitigates tissue damage, reduces bacterial burden, and facilitates microbiota recovery during *C. difficile* infection [57,121,122] (Fig. 3D). These diametrically opposed effects perfectly illustrate the double-edged nature of CGRP as an “immune orchestrator”.

The loss of intestinal epithelial integrity, compromised mucosal barrier function, and aberrant immune responses are primary instigators of inflammatory bowel disease [123,124]. Studies reveal that in dextran sulfate sodium (DSS)-induced colitis models, mice with chemogenetically silenced or resiniferatoxin (RTX)-ablated TRPV1^+^ nociceptors exhibit exacerbated inflammatory responses and defective tissue repair compared to their littermate controls. Notably, although the ablated group shows more severe neutrophil infiltration in the colon, the proportions of IL-17A- and IFN-γ-secreting CD4^+^ T cells, as well as regulatory T cells (Tregs), remain comparable between the 2 groups. This indicates that in this model, TRPV1^+^ nociceptors are pivotal in modulating intestinal tissue damage, inflammation, and repair, without markedly impacting the adaptive immune response [110].

Parallel conclusions were drawn in another study: In the DSS-induced colitis model, mice deficient in Nav 1.8-expressing nociceptors (via diphtheria toxin fragment A) display more severe colonic symptoms compared to controls, characterized by pronounced weight loss, shortened colon length, extensive disruption of colonic architecture, increased immune cell infiltration, and goblet cell depletion [125]. Collectively, these findings suggest that the activation of nociceptive sensory neurons confers protection against colitis via the CGRP–RAMP1 signaling axis, a mechanism likely mediated by the regulation of intestinal mucus secretion and the gut microbiota.

The intestinal barrier consists of epithelial cells overlaid by a mucus layer rich in mucin-2. This mucus layer shields the gut from autodigestion by gastric acid, digestive enzymes, and bile salts, while simultaneously serving as a physical barricade against pathogen invasion [126,127]. Goblet cells, specialized intestinal epithelial cells, synthesize mucins and associated proteins to form this protective layer, safeguarding tissues from pathogenic breaches [128]. In the colon, nociceptors regulate goblet cell mucus secretion via the CGRP–RAMP1 axis, thereby mediating barrier protection. Nav1.8^+^ neurons residing adjacent to colonic goblet cells are critical regulators of mucus secretion. Ablation of these neurons results in a thinned colonic mucus layer; conversely, specific activation of Nav1.8^+^ neurons—either chemogenetically via designer receptors exclusively activated by designer drugs or via capsaicin-induced nociceptor activation—triggers rapid mucus secretion from goblet cells, a process completely abrogated by RAMP1 antagonists. Mechanistically, CGRP released from Nav1.8^+^ neurons directly acts on RAMP1 expressed on goblet cells to mediate mucin synthesis and secretion [125] (Fig. 3E). This CGRP-based mucus regulation mechanism, in concert with the aforementioned VIP-ILC3-mediated antimicrobial defense system, coconstructs a robust dual physical-immune barrier.

Interestingly, intestinal CGRP synthesis is markedly reduced in germ-free (GF) mice compared to specific-pathogen-free controls. Following fecal microbiota transplantationfrom specific-pathogen-free donors to GF recipients, colonic CGRP release is up-regulated. This process is strictly nociceptor-dependent, as RTX-mediated nociceptor ablation prior to fecal microbiota transplantation abolishes this up-regulation. Furthermore, antibiotic treatment in normal mice also decreases colonic CGRP release, whereas this treatment has no effect on Nav1.8^+^ nociceptor-deficient mice. These data demonstrate that the gut microbiota actively regulates CGRP secretion from Nav1.8^+^ neurons [125].

Conversely, CGRP release reciprocally shapes the composition of the gut microbiota. Ablation of TRPV1^+^ nociceptors, while not markedly altering overall microbial diversity, reduces the relative abundance of *Muribaculaceae* (phylum Bacteroidetes) and *Ruminococcaceae* (phylum Firmicutes), while concomitantly increasing the relative abundance of *Turibacteriaceae* (phylum Firmicutes) and *Erysipelotrichaceae* [110,125]—taxa potentially implicated in mediating intestinal inflammation [129–131] (Fig. 3F). This microbial dysbiosis is transmissible: When intestinal contents from dimethyl sulfoxide- or RTX-treated mice are transplanted into GF mice, the recipients phenocopy the donor’s microbial abundance profile. Furthermore, GF mice receiving microbiota from RTX-treated donors exhibit exacerbated symptoms in the DSS-induced colitis model. This result underscores that nociceptors exert colonic protection by modulating the gut microbiota [110].

Further investigation identified vancomycin-sensitive gram-positive bacteria as key drivers of exacerbated intestinal inflammation following nociceptor ablation. Vancomycin treatment (which depletes gram-positive bacteria) restores the tolerance of RTX-treated mice to DSS back to the levels of the dimethyl sulfoxide-treated group. In contrast, neomycin treatment (depleting gram-negative bacteria) further increases the susceptibility of RTX-treated mice to DSS. In nociceptor-ablated GF mice, colonization with *Clostridium* exacerbates intestinal inflammation; remarkably, in wild-type GF mice, *Clostridium* colonization confers tissue protection. This indicates an intimate and intricate interplay between TRPV1^+^ nociceptors and specific enteric bacteria, collaboratively orchestrating intestinal inflammation and tissue protection [110,132]. These discoveries suggest the existence of a highly precise, dynamic equilibrium network among the nervous system, immune system, and microbiota.

In conclusion, the neuropeptide network—comprising the proinflammatory drive of SP, the barrier-stabilizing effects of VIP, and the microenvironment-dependent, bidirectional regulation by CGRP—serves as the core hub connecting neural sensing with mucosal immunity. This intricately balanced triad also raises a critical caveat for clinical translation: Prolonged or systemic pharmacological blockade of CGRP signaling (such as with anti-CGRP therapeutics currently widely employed for migraine management) may inadvertently disrupt the delicate counterbalances among SP, VIP, and CGRP. Such disruption could destabilize the fragile equilibrium of the intestinal microbiota and mucus barrier, thereby elevating the potential risk of developing intestinal inflammation, microbial dysbiosis, or opportunistic infections in susceptible patient cohorts.

### The neuroimmune CGRP–RAMP1 axis regulates skin barrier protection and wound healing

Sensory neuron innervation in the skin forms an elaborate neural network, connecting the epidermis, dermis, and subcutaneous tissue to the CNS via peripheral nerve fibers [133]. These neurons, primarily originating from the DRG, encompass various subtypes, including TRPV1^+^ nociceptive neurons and mechanosensory neurons [134]. Their nerve endings distribute across all skin layers, forming close contacts with keratinocytes and branching networks around hair follicles and blood vessels. Upon activation, this intricate innervation regulates the local microenvironment through the release of a neuropeptide matrix, notably SP, VIP, and CGRP [134]. Within this matrix, SP classically functions as a potent proinflammatory amplifier and pruritogen, whereas VIP primarily acts as a homeostatic stabilizer that mitigates excessive inflammation [135,136]. In contrast, CGRP operates as a highly microenvironment-dependent “immune orchestrator”, exhibiting dual regulatory functions that profoundly shape skin regeneration, microbial tolerance, and the trajectory of inflammatory diseases.

**Fig. 4.**
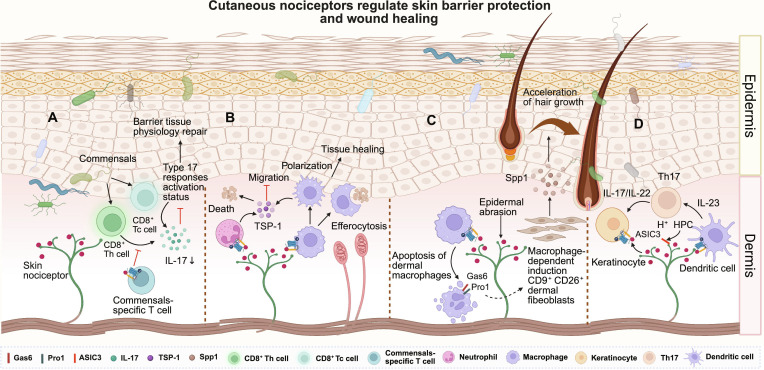
Cutaneous nociceptors regulate skin barrier protection and wound healing through the calcitonin gene-related peptide (CGRP)-receptor activity-modifying protein 1 (RAMP1) axis. (A) CGRP acts on RAMP1 expressed by skin commensal-specific T cells, inhibiting interleukin-17 (IL-17) production (IL-17↓) by CD8^+^ Tc cells and CD8^+^ Th cells, which promotes physiological repair of the tissue barrier. (B) CGRP released by sensory neurons induces neutrophils and macrophages to release thrombospondin-1 (TSP-1) (TSP-1↑) and promotes macrophage polarization. TSP-1, in turn, inhibits the migration of neutrophils and macrophages while accelerating cell apoptosis, which accelerates tissue wound healing. (C) Epidermal abrasion induces the activation of cutaneous nociceptors and the release of CGRP (CGRP↑). CGRP acts on specific receptors on macrophages, inducing macrophage apoptosis and increasing the proportion of CD9^+^CD26^+^ fibroblasts (CD9^+^CD26^+^ fibroblasts↑). Secreted phosphoprotein 1 (Spp1) secreted by CD9^+^CD26^+^ fibroblasts promotes hair growth. (D) In the psoriasis model, H^+^ and LPC can activate acid-sensing ion channels (ASICs) on sensory neurons, which subsequently triggers the release of CGRP. CGRP acts on dendritic cells (DCs) and keratinocytes, positively regulating T helper 17 (Th17)-type inflammatory responses, thereby exacerbating skin inflammation in psoriasis. This figure was created in BioRender. Gao, C. (2026) https://BioRender.com/pyktjif (agreement number: TF29I8ZL6P)

The skin, as the largest barrier organ, possesses remarkable regenerative capacity. Damage to the skin barrier initiates a wound healing cascade requiring the coordinated responses of immune cells, the extracellular matrix, epithelial cells, and the microbiota [137,138]. During this process, neuropeptides exert divergent yet complementary effects. While VIP accelerates tissue repair through proangiogenic and anti-inflammatory pathways [136], the chronic hypersecretion of SP often impairs healing, contributing to nonhealing wounds [135]. Distinctively, CGRP delicately fine-tunes the balance between rapid closure and immune tolerance. As a primary site for microbial colonization, the skin relies on commensal bacteria to induce the up-regulation of the CGRP receptor RAMP1 on T cells. The CGRP–RAMP1 signaling axis suppresses commensal-induced type 17 responses (mediated by type 17 helper T [Th17] and cytotoxic T [Tc17] cells), altering the transcriptional profile of keratinocytes and subsequently modulating the pace of epithelial wound repair to maintain immune tolerance [77] (Fig. 4A). Conversely, in chronic nonhealing scenarios like diabetic peripheral neuropathy, CGRP actively drives tissue repair and inflammation resolution. Engineered CGRP acts on neutrophils and macrophages via RAMP1, inducing the release of thrombospondin-1. Thrombospondin-1 not only accelerates neutrophil efferocytosis but also, in the presence of IL-4/IL-13 or IL-10, promotes macrophage polarization toward an anti-inflammatory, repair-promoting M2 phenotype, thereby accelerating wound healing and muscle regeneration [40] (Fig. 4B).

Beyond wound repair, the neuroimmune axis profoundly governs the regeneration of tissue appendages. Following epidermal abrasion, activated TRPV1^+^ nociceptors secrete CGRP, which binds to the CALCRL–RAMP1 complex on dermal macrophages, initiating macrophage apoptosis. These apoptotic macrophages release growth arrest-specific 6 (Gas6) and protein S (Pros1). These ligands bind to the Axl receptor on dermal fibroblasts, driving their massive expansion into a CD9^+^CD26^+^ phenotype. These expanded fibroblasts secrete secreted phosphoprotein 1, which prompts quiescent hair follicle stem cells to proliferate, thereby initiating robust hair regeneration [139] (Fig. 4C). In stark contrast to CGRP’s regenerative cascade, SP exhibits diametrically opposed effects in the hair follicle, often inducing premature catagen (regression) and inhibiting hair shaft elongation through neurogenic inflammation [140].

However, in pathological inflammatory states such as psoriasis, the dual nature of CGRP shifts from a repair-promoting factor to a potent proinflammatory driver, synergizing with SP. Psoriasis is heavily driven by neurogenic inflammation and the IL-23/IL-17 axis [141–143]. While SP exacerbates psoriatic lesions via robust mast cell degranulation [135], CGRP levels and its receptor expression are concurrently elevated in the dermal DCs of psoriasis patients [144–146]. Mechanistically, CGRP released from activated nociceptors acts on dermal DCs, driving the up-regulation of pro-IL-1β and IL-23 independent of other inflammatory signals. This nociceptor-DC crosstalk subsequently dictates IL-17A production by γδ T cells [147,148]. Furthermore, the activation of the sensory acid-sensing ion channel 3 by acid and lysophosphatidylcholine induces CGRP release, which not only promotes DC-derived IL-23 but also directly induces keratinocyte proliferation and epidermal thickening [149,150] (Fig. 4D).

Collectively, the cutaneous neuroimmune landscape is maintained by the delicate interplay of SP’s proinflammatory drive, VIP’s barrier-stabilizing effects, and CGRP’s microenvironment-dependent duality. This complex equilibrium raises a critical clinical caveat: Long-term or systemic pharmacological blockade of CGRP signaling, such as with anti-CGRP therapeutics widely used for migraine management, may inadvertently disrupt this neural-immune-microbiota triad in the skin. Such disruptions could elevate the risk of impaired wound healing, altered microbial tolerance, or exacerbated dermatological conditions in susceptible patient populations.

### CGRP regulates pulmonary immune homeostasis

The respiratory tract, serving as a vital mucosal interface exposed continuously to airborne antigens and pathogens, relies heavily on a dense network of sensory and autonomic nerve fibers to maintain barrier integrity. Upon exposure to environmental insults, these neural circuits release a diverse matrix of neuropeptides, most notably SP, VIP, and CGRP [151,152]. Within this triad, SP and VIP often represent functional extremes: SP classically acts as a potent proinflammatory amplifier and bronchoconstrictor, while VIP serves as a critical homeostatic stabilizer and bronchodilator [153,154]. CGRP, however, transcends the role of a unidirectional switch; it operates as a highly context-dependent “immune orchestrator”. By dynamically modulating immune cell activity and epithelial survival, CGRP can either amplify early inflammatory cascades or execute specific signaling programs to restrain excessive tissue damage, positioning it as a central regulatory node in pulmonary conditions ranging from allergic asthma to acute lung injury (ALI).

**Fig. 5.**
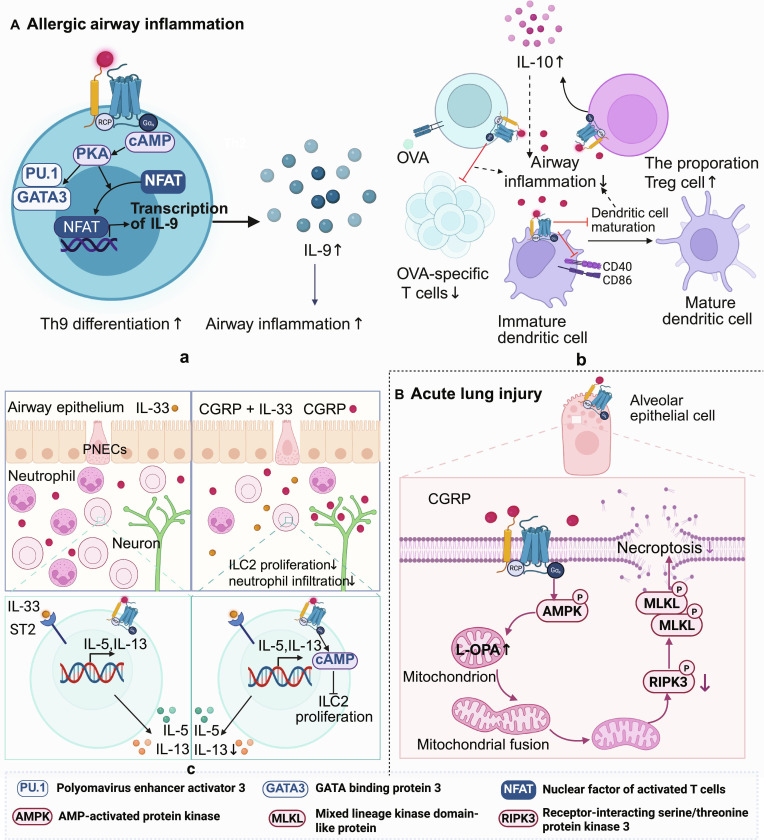
Calcitonin gene-related peptide (CGRP) is involved in the regulation of pulmonary immune homeostasis. (A) a. Acting on Th9 cells via the cyclic adenosine monophosphate (cAMP)/protein kinase A (PKA) pathway, CGRP induces the expression of GATA3 and PU.1 and the production of interleukin-9 (IL-9) (IL-9↑), promoting Th9 differentiation and exacerbating airway inflammation. b. CGRP reduces the proportion of ovalbumin (OVA)-specific T cells (OVA-specific T cells↓), increases the proportion of Treg cells (Treg cells↑), inhibits dendritic cell maturation (dendritic cell maturation↓), suppresses IL-13 production (IL-13↓) and proliferation of group 2 innate lymphoid cells (ILC2s) (ILC2 proliferation↓), thereby alleviating airway inflammation. c. CGRP activates the cAMP signaling pathway by binding to receptor activity-modifying protein 1 (RAMP1) receptor to negatively regulate ILC2 responses, thereby inhibiting airway inflammation. (B) In acute lung injury, CGRP protects alveolar epithelial cells by activating the AMP-activated protein kinase (AMPK)/long form of optic atrophy 1 (L-OPA1) pathway to improve lipopolysaccharide (LPS)-induced mitochondrial dysfunction. This figure was created in BioRender. Gao, C. (2026) https://BioRender.com/50fqrw6 (agreement number: ZK29I8ZKHY)

In the pathogenesis of allergic airway inflammation, the functional output of this neuropeptide matrix is highly dependent on the cellular target and the prevailing cytokine milieu. SP aggressively exacerbates asthmatic responses by binding to the NK-1R on epithelial and immune cells, triggering robust neurogenic inflammation, mucus hypersecretion, and airway hyperresponsiveness [153]. In certain spatial or temporal contexts, CGRP mirrors this proinflammatory drive by actively promoting the differentiation of Th9 cells. Mechanistically, CGRP signaling via the cAMP/PKA pathway up-regulates the transcription factors PU.1 and GATA3 in Th9 cells, induces the nuclear translocation of NFATc2, and inactivates glycogen synthase kinase-3 beta, collectively driving robust IL-9 production [155,156] (Fig. 5A, a). The essential nature of this axis is highlighted in T cell-specific *Ramp1*^−/−^ mice, which exhibit markedly reduced pulmonary inflammatory infiltration following ovalbumin challenge. Furthermore, elevated CGRP expression in the sensory nerves of patients with fatal asthma corroborates this pathogenic role [157]. Concurrently, CGRP can cooperate with epithelial-derived alarmins (IL-25 and IL-33) to activate ILC2s, augmenting IL-5 production and amplifying eosinophilia [155].

Paradoxically, in different phases of the same allergic response, CGRP deploys robust anti-inflammatory mechanisms to prevent mucosal damage—a protective trait it shares with VIP. While VIP profoundly suppresses type 2 inflammation and promotes Treg functions to maintain airway tolerance [154], CGRP actively regulates the initiation phase of allergy by inhibiting the maturation of pulmonary DCs. Following allergen exposure, CGRP blocks the LPS-induced up-regulation of costimulatory molecules (CD86 and CD40) on bone marrow-derived DCs. Consequently, these tolerogenic DCs inhibit ovalbumin-specific T cell proliferation, suppress proinflammatory TNF-α secretion, and reciprocally expand the Treg pool while boosting IL-10 production [158,159] (Fig. 5A, b). Intriguingly, upon sustained receptor binding, CGRP also serves as a negative feedback regulator for ILC2s. It suppresses IL-33-induced ILC2 expansion and type 2 cytokine (IL-5 and IL-13) secretion in a dose-dependent manner. The absence of this negative regulatory loop in Ramp1 knockout mice leads to unbridled ILC2 proliferation and exacerbated eosinophilia [160] (Fig. 5A, c).

In contrast to their multifaceted roles in chronic allergy, these factors play protective roles in ALI as critical tissue-survival factors. During severe pulmonary insults, SP often aggravates the early acute phase by promoting massive neutrophil influx and vascular leakage [161]. In stark contrast, CGRP directly safeguards alveolar epithelial cells from necroptosis by activating the AMP-activated protein kinase pathway. This activation up-regulates the long form of optic atrophy 1, promoting mitochondrial fusion and restoring metabolic function to maintain epithelial viability [162] (Fig. 5B). Concurrently, the CGRP/RAMP1 signaling axis—much like VIP’s suppressive effect on macrophage inflammatory pathways—systemically dampens the hyperactivation of alveolar macrophages, monocyte-derived macrophages, and recruited neutrophils, thereby curtailing the lethal cytokine storm characteristic of ALI [163]. The physiological necessity of this neuropeptide in acute defense is further confirmed by the administration of the CGRP receptor antagonist CGRP (8-37), which markedly exacerbates LPS-induced lung injury [164].

Collectively, the pulmonary neuroimmune landscape is governed by the delicate counterbalance of SP’s proinflammatory drive, VIP’s barrier-stabilizing effects, and CGRP’s microenvironment-dependent duality. This intricate functional divergence raises a critical clinical caveat: The long-term systemic blockade of CGRP pathways may inadvertently disrupt this finely tuned neural-immune rheostat. Such pharmacological interventions risk stripping the lungs of vital compensatory and tissue-protective mechanisms, potentially predisposing susceptible individuals to exaggerated ALIs or fundamentally worsening the trajectory of preexisting airway inflammatory diseases.

## Calcitonin Gene-Related Peptide: A Pivotal Regulator of Tumor Growth and Adaptive Immunity

The TME engages in complex, bidirectional crosstalk with the peripheral nervous system. Rather than being passive bystanders, tumor cells actively “hijack” local neural circuits, co-opting neural-derived mediators to dynamically regulate proliferation, invasion, metastasis, and inflammatory responses [165,166]. Within this intricate neuroneoplastic web, a triad of neuropeptides-SP, VIP, and CGRP-has emerged as paramount. While SP classically fuels tumor proliferation and neural invasion, and VIP acts as a potent immunosuppressive checkpoint, CGRP functions as a highly microenvironment-dependent orchestrator. Depending on the spatial context and the specific target cells, CGRP dictates a multifaceted regulatory network that heavily influences cancer trajectories and systemic adaptive immunity (Table 1).

**Table 1.** The molecular mechanisms by which CGRP directly or indirectly affects cancer development

Type of tumor	Target cell	The impact on tumor	The molecular mechanism of tumor immune suppression
Melanoma	CD8^+^ T cell	Promote tumor growth	• Increase the expression of immune checkpoint receptors (PD-1, LAG3, and TIM3) on CD8^+^ T cells [36].
			• Reduce the secretion of IFN-γ, TNF-α, and IL-2 [36].
			• Hinder the formation of TLS [168].
Head and neck squamous cell carcinoma (HNSCC)	CD4^+^ T cell CD8^+^ T cell	Promote tumor growth	• Inhibit the activation of CD4^+^ T cell and CD8^+^T cell [171].
Medullary thyroid carcinoma (MTC)	DC	Promote tumor growth	• Suppression of KLF2 loss in DCs via the cAMP-KLF2 pathway [175].
			• DC dysfunction, characterized by decreased antigen presentation and costimulatory capacity, leads to reduced activation of CD8^+^ T cells [175].
Oral squamous cell carcinoma (OSCC)	OSCC cell	Promote tumor growth and metastasis	• Reduce tumor immune cell infiltration: CD4^+^ T cell, CD8^+^T cell, and NK1.1^+^ NK cell [173].
			• Induce cytoprotective autophagy by activating Rap1-GTPase signaling [180].
Gastric cancer (GC)	GC cell	Promote tumor growth and metastasis	• Up-regulates the Rb-E2F signaling pathway by activating the PI3K-AKT and CaMK pathways in gastric cancer cells [182].
			• Promote tumor cell proliferation and survival [182].

CGRP, calcitonin gene-related peptide; DC dendritic cell; cAMP, cyclic adenosine monophosphat; NK, natural killer; IFN-γ, interferon-γ; TNF-α, tumor necrosis factor-α; IL-2, interleukin-2; PI3K, phosphoinositide 3-kinase; TLS, tertiary lymphoid structure; KLF2, Krüppel-like factor 2; GTPase, guanosine triphosphatase; PD-1, programmed cell death protein 1

### VIP and CGRP: Orchestrators of T cell exhaustion and immune evasion

A primary mechanism by which tumors secure their survival is by co-opting neuropeptides to suppress T cell-mediated antitumor immunity. VIP serves as a classic neuroimmune checkpoint in this regard. In highly desmoplastic microenvironments like pancreatic ductal adenocarcinoma, tumor and stromal cells overexpress VIP. This neuropeptide binds to VPAC receptors on T cells, directly dampening their activation. Pharmacological blockade of VIP signaling synergizes profoundly with anti-programmed cell death protein 1 (PD-1) therapy to restore cytotoxic T cell function [167].

Parallel to VIP, CGRP enforces immune evasion, albeit through distinct exhaustion pathways. In melanoma, elevated sensory innervation (marked by TRPV1 and Nav1.8 expression) correlates with a poor clinical prognosis [36]. Melanoma cells actively promote TRPV1^+^ neurite outgrowth and enhance neuronal excitability by up-regulating *Calca* and *TrkA* expression and secreting secretory leukocyte protease inhibitor. Neural-derived CGRP then binds directly to the the CLR/RAMP1 receptor complex on cytotoxic CD8^+^ T cells. Instead of activating these cells, the CGRP–RAMP1 axis initiates a profound exhaustion program: It up-regulates inhibitory checkpoint receptors (PD-1, LAG3, and TIM3) while suppressing the production of critical effector cytokines (IFN-γ, TNF-α, and IL-2) [36]. While interventions like TRPV1^+^ neuron ablation or RAMP1 antagonism restore immunity, optogenetic activation accelerates progression [36]. Interestingly, while some studies indicate sensory nerves suppress immunity by hindering tertiary lymphoid structure formation [168], other conflicting reports highlight the extreme heterogeneity of the TME [169,170].

**Fig. 6.**
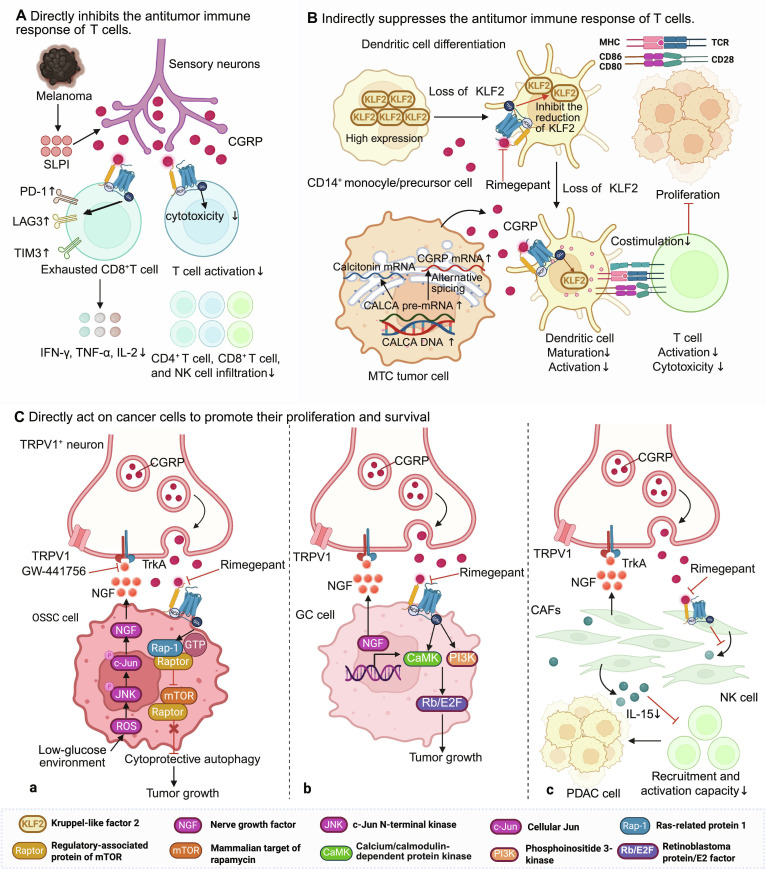
Calcitonin gene-related peptide (CGRP)-mediated regulation of cancer immune surveillance. (A) Nociceptor neurons are activated by secretory leukocyte protease inhibitor (SLPI) secreted by melanoma and secrete CGRP, which acts on CD8^+^ T cells to increase the expression of immune checkpoints and promote CD8^+^ T cell exhaustion. In head and neck squamous cell carcinoma, nerves within the tumor express CGRP, which acts on receptors on the surface of T cells to inhibit T cell activation and the cytotoxic effect of CD8^+^ T cells on tumor cells. (B) In medullary thyroid carcinoma (MTC), CGRP secreted by MTC tumor cells directly acts on dendritic cells (DCs), inhibiting the development and function of DCs through the AMP-Krüppel-like factor 2 (KLF2) pathway, thereby indirectly affecting the activation of T cell antitumor immunity. (C) a. Oral squamous cell carcinoma cells increase the expression of nerve growth factor (NGF) through the JNK/c-Jun pathway (NGF↑) in a low-glucose environment, and the secreted NGF induces transient receptor potential vanilloid 1 (TRPV1)^+^ neurons to produce CGRP. b. CGRP activates the Rap1-guanosine triphosphatase (GTPase) signaling pathway, thereby competitively inhibiting the interaction between Raptor and mechanistic target of rapamycin (mTOR), inducing protective autophagy in cancer cells and promoting tumor growth. Gastric cancer cells highly express NGF, which promotes the growth of CGRP-positive nociceptive neurons into tumors and their release of CGRP through the NGF-tropomyosin receptor kinase A (TrkA) axis. CGRP binds to the receptor activity-modifying protein 1 (RAMP1) receptor on gastric cancer cells, activating the phosphoinositide 3-kinase (PI3K)-AKT and CaMK pathways to promote E2F activity (E2F activity↑), thereby driving tumor progression. c. In the pancreatic ductal adenocarcinoma microenvironment, NGF released by cancer-associated fibroblasts (CAFs) promotes the synthesis and release of calcitonin CGRP in nociceptive neurons through TrkA signaling (CGRP↑). CGRP binds to the RAMP1 receptor on the surface of CAFs, inhibits the secretion of interleukin-15 (IL-15) (IL-15↓), and weakens the recruitment and activation of NK cells. Meanwhile, nociceptive neurons and CAFs form an NGF–CGRP positive feedback loop, jointly promoting tumor progression and cancer pain. This figure was created in BioRender. Gao, C. (2026) https://BioRender.com/ryh767w (agreement number: JI29I9PB86)

This CGRP-mediated immune evasion extends to head and neck squamous cell carcinoma and oral squamous cell carcinoma (OSCC), where perineural invasion correlates with fatal outcomes [171]. Strikingly, tumor cells can also secrete factors like SLIT2 to co-opt inter-organ neuroimmune circuits, prompting sensory nerves to release CGRP that actively reshapes the tumor-draining lymph nodes into an immunosuppressive state [172]. Locally, elevated CGRP directly curtails the cytolytic capacity of CD8^+^ T cells and reduces IFN-γ expression. Conversely, denervation or administration of CGRP receptor antagonists (e.g., BIBN4096) profoundly increases the infiltration of activated CD4^+^ (Th1) and CD8^+^ T cells while reducing the Treg population, synergizing with radiotherapy to inhibit tumor growth [170,173]. Furthermore, clinical data reveal that high RAMP1 expression in the TME serves as a negative predictive biomarker for immune checkpoint inhibitors [174]. Calca knockout models demonstrate markedly increased intratumoral accumulation of CD4^+^, CD8^+^ T cells, and NK cells, confirming that CGRP fundamentally restricts effector immune cell infiltration [173] (Fig. 6A).

### CGRP indirectly remodels the TME via antigen-presenting cells

Beyond directly exhausting T cells, CGRP orchestrates systemic immune tolerance by corrupting the function of professional antigen-presenting cells. A striking example is observed in medullary thyroid carcinoma, where tumor cells themselves aberrantly express high levels of *CALCA* and establish an autocrine/paracrine CGRP loop [175–178]. Medullary thyroid carcinoma-derived CGRP binds to receptors on local DCs, activating the cAMP signaling pathway to block the developmental down-regulation of Krüppel-like factor 2 (KLF2). By sustaining abnormally high KLF2 levels, CGRP arrests DCs in a dysfunctional, immature state characterized by drastically reduced expression of essential costimulatory molecules (CD80, CD86, and CD40). Consequently, these tolerogenic DCs fail to effectively prime CD8^+^ T cells. This pathogenic mechanism starkly contrasts with benign papillary thyroid carcinoma, where KLF2 expression naturally declines as DCs mature. In vitro interventions using CGRP receptor antagonists (like Rimegepant) or the cAMP inhibitor SQ22536 successfully restore KLF2 degradation and rescue DC function, establishing the CGRP–cAMP–KLF2 axis as a critical driver of DC dysfunction [175] (Fig. 6B).

### SP and CGRP: Drivers of cancer cell proliferation, survival, and stromal remodeling

In addition to their immunomodulatory roles, neuropeptides act directly on cancer cells and stroma as potent survival, proliferative, and invasive signals. SP is a notorious driver of this aggressive phenotype. In pancreatic cancer, SP released from local primary sensory nerves binds to the NK-1R heavily overexpressed on cancer cells. This SP/NK-1R signaling accelerates tumor proliferation and specifically drives cancer cells to aggressively invade the perineural space, facilitating local recurrence and severe cancer-associated pain [179].

Working in tandem with the invasive drive of SP, CGRP ensures metabolic survival. In OSCC, hypoglycemic conditions trigger tumor cells to generate reactive oxygen species, activating the JNK-c-Jun pathway to secrete NGF. NGF binds to TrkA receptors on adjacent nociceptive endings, inducing robust CGRP release. This secreted CGRP engages CLR/RAMP1 receptors directly on OSCC cells, activating Rap1-guanosine triphosphatase signaling to disrupt mechanistic target of rapamycin–Raptor interactions. This induces cytoprotective autophagy, allowing tumor cells to survive severe energy deprivation. Clinically, this vicious cycle correlates with high Ki67^+^ proliferating indices, robust perineural invasion, and lymph node metastasis [180,181] (Fig. 6C, a).

A parallel NGF–CGRP axis drives tumor progression in the gastric cancer (GC) microenvironment. GC cells overexpress NGF to preferentially attract CGRP^+^ peptidergic nociceptors originating from the jugular nerve complex and DRG T7-T13. Upon NGF-TrkA-mediated release, CGRP binds to RAMP1 on GC cells, activating the phosphoinositide 3-kinase-AKT and CaMK pathways to markedly enhance E2F transcription factor activity. Chemogenetic activation of these nociceptors accelerates GC proliferation and metastasis, while pharmacological ablation substantially restricts tumor growth, reduces circulating tumor cells, and limits liver metastases [182,183] (Fig. 6C, b). Strikingly, recent evidence indicates that in certain gastrointestinal cancers, tumor cells themselves can intrinsically secrete CGRP and express RAMP1, establishing a robust autocrine growth loop entirely independent of nerve fibers [184].

Finally, CGRP manipulates the tumor stroma. In pancreatic ductal adenocarcinoma, an NGF–CGRP positive feedback loop forms between nociceptors and cancer-associated fibroblasts (CAFs). NGF released by CAFs triggers neuronal CGRP secretion, which reciprocally binds to RAMP1 on CAFs to suppress their production of IL-15. This deprivation severely impairs the recruitment and activation of NK cells. Disrupting this axis with Rimegepant yields both potent antitumor (by restoring IL-15/NK cell function) and analgesic effects [185] (Fig. 6C, c).

### The broader context: Bidirectional modulation of adaptive immunity

**Fig. 7.**
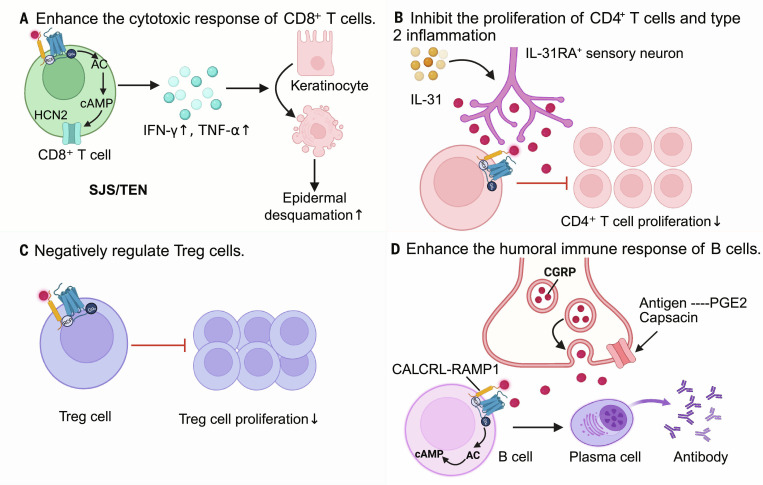
Calcitonin gene-related peptide (CGRP)-mediated regulation of adaptive immune response. (A) In Stevens–Johnson syndrome (SJS) and toxic epidermal necrolysis (TEN), CGRP acts on the calcitonin receptor-like receptor (CALCRL)-receptor activity-modifying protein 1 (RAMP1) receptor on CD8^+^ T cells to enhance the cytotoxic response of CD8^+^ T cells (CD8^+^ T cell cytotoxicity↑), induce keratinocyte apoptosis, and aggravate epidermal detachment. (B) In allergic dermatitis, CGRP inhibits CD4^+^ T cell proliferation and type 2 inflammatory responses. (C) CGRP secreted after transient receptor potential vanilloid 1 (TRPV1)^+^ neuron activation inhibits Treg cell proliferation. (D) CGRP secreted by splenic sensory neurons acts on the CALCRL–RAMP1 receptor on B cells to promote germinal center reactions through an adenylate cyclase-dependent pathway. This figure was created in BioRender. Gao, C. (2026) https://BioRender.com/fhu1odj (agreement number: TX29MPHK1Y)

To fully appreciate this neuroimmune matrix, it must be viewed within the broader context of adaptive immunity, where neuropeptide function is highly plastic. Beyond its established role in enhancing CD8^+^ T cell cytotoxicity against *LCMV* infection, CGRP exhibits profound context-dependent immunomodulation. In Stevens–Johnson syndrome (SJS) and toxic epidermal necrolysis (TEN), CGRP binding to RAMP1 on CD8^+^ T cells activates the hyperpolarization-activated cyclic nucleotide-gated channel 2. This massively augments T cell cytotoxic activity, exacerbating keratinocyte apoptosis and epidermal detachment [186] (Fig. 7A). Conversely, in atopic dermatitis, IL-31-stimulated IL31RA^+^ sensory neurons release CGRP to suppress CD4^+^ T cell proliferation and type 2 cytokine production. Genetic ablation of *Il31ra* intensifies type 2 inflammation, explaining the paradoxical dermatitis observed during anti-IL31RA therapy [35] (Fig. 7B).

CGRP also exerts precise spatial control over mucosal immune tolerance. In the gut, chemogenetic activation (via a Cre recombinase-driven designer receptors exclusively activated by designer drugs system) of TRPV1^+^ nociceptors in the DRG prompts local CGRPα release. This binds directly to the RAMP1–CALCRL complex on (RAR-related orphan receptor γ-positive) Tregs in the colon and cecum, profoundly remodeling their transcriptome (e.g., up-regulating heat shock protein genes) and inhibiting their proliferation. This suppression weakens intestinal barrier protection, increasing host susceptibility to DSS-induced colitis and *Citrobacter rodentium* infection [106] (Fig. 7C).

Conversely, transitioning from cellular immunosuppression in the gut to humoral enhancement in lymphoid organs, splenic sensory neuron-derived CGRP is essential for germinal center reactions. Capsaicin- or antigen-induced prostaglandin E2 activates specific nociceptors from the left T8-T13 DRG, triggering targeted CGRP release into splenic B cell zones. CGRP-CALCRL/RAMP1 signaling in B cells orchestrates germinal center formation. Nociceptor ablation drastically reduces antigen-specific germinal center B cells, memory B cells, and high-affinity IgG/IgM titers. This absolute requirement is confirmed in *Calcrl^−/−^* and *Ramp1^−/−^* mice, whose severely attenuated humoral responses can be entirely rescued by bypassing the CGRP receptor via forskolin-mediated direct activation of adenylate cyclase and cAMP elevation [100] (Fig. 7D)

Conclusions and Clinical Implications: Collectively, these findings redefine the neuroneoplastic triad of SP, VIP, and CGRP not merely as pain neurotransmitters, but as master regulators of tissue homeostasis, adaptive immunity, and TME remodeling. Tumors exhibit a phenomenon akin to “neural addiction”, co-opting these neuropeptide pathways to secure metabolic survival, drive physical invasion, and enforce immune evasion [187]. However, the profound context dependency of these immunomodulatory effects raises appreciable clinical considerations. While targeting the VIP–VPAC or CGRP–RAMP1 axes presents a highly promising strategy to overcome immune checkpoint inhibitor resistance and halt tumor progression, it is imperative to proceed with caution. The systemic and long-term pharmacological blockade of these pathways (e.g., anti-CGRP therapies currently utilized in chronic migraine management) may inadvertently disrupt the delicate neuroimmune balance in diverse organ systems. Such disruptions could potentially compromise essential humoral responses to infections or exacerbate latent autoimmune and inflammatory conditions, underscoring the critical need for locally targeted or carefully titrated therapeutic interventions.

## Discussion

### Context-dependent mechanisms of CGRP signaling heterogeneity

The pleiotropic actions of CGRP across disease contexts are not stochastic but governed by 3 hierarchically organized contextual determinants, collectively underpinning the functional plasticity of the CGRP–NIHS Axis.

### Receptor subtype selectivity and cell-type-specific signaling modules

CGRP elicits divergent biological outcomes through differential engagement of CALCRL-RAMP complexes. CALCRL–RAMP1, predominantly expressed on myeloid cells (macrophages, neutrophils, DCs), propagates immunosuppressive signals via NLRP3 inflammasome inhibition [68] and M2 polarization [67]. In contrast, CALCRL–RAMP3 enrichment on lymphocytes (T/B cells) promotes Th1 differentiation [101] and germinal center formation [100]. Strikingly, even within the same cell lineage, context-specific signaling adaptors rewire CGRP function: In CD8^+^ T cells from SJS/TEN, CGRP–RAMP1 couples with stromal interaction molecule 1 to enhance hyperpolarization-activated cyclic nucleotide-gated channel 2-dependent cytotoxicity [186], whereas in tumor-infiltrating CD8^+^ T cells, the identical receptor complex recruits fas-associated protein with death domain (FADD) to up-regulate PD-1/LAG3, driving T cell exhaustion [36]. This adaptor-switching paradigm represents a previously unrecognized layer of signaling diversification.

### Microenvironmental signal integration

The functional output of CGRP is dynamically sculpted by tissue microenvironment cues. In bacterial infection settings, CGRP synergizes with LPS/TNF-α to suppress neutrophil chemotaxis [48], whereas in β-glucan-rich fungal niches, it cooperates with Dectin-1-PLCγ signaling to license DC IL-17 production [41]. Within the hypoxic, NGF-enriched TME, CGRP establishes a feedforward loop with CAFs to repress IL-15 secretion, blunting NK cell activation [185]. Conversely, in commensal bacteria-replete intestinal mucosa, CGRP sustains barrier integrity through RORγ^+^ Treg modulation [106]. These observations underscore CGRP as a “signal integrator” that translates microenvironmental inputs into context-appropriate immune responses.

### Isoform-specific functional specialization

α- and β-CGRP exhibit nonredundant roles in the CGRP–NIHS Axis. α-CGRP, predominantly expressed in DRG and CNS, mediates T cell exhaustion in melanoma [42] and Th2-driven airway inflammation in asthma [173,175]. β-CGRP, the dominant peripheral isoform, regulates gut microbiota composition [121,137] and coordinates type 2 immunity against helminths [90]. Genetic validation studies confirm this dichotomy: Calca (α-CGRP) ablation exacerbates viral infections via CD8^+^ T cell dysfunction [109], while Calcb (β-CGRP) deletion disrupts intestinal barrier homeostasis [121,137].

### From context dependency to clinical translation: The “corticosteroid-like” dilemma of systemic blockade

Therapeutic targeting of the CGRP pathway (e.g., via monoclonal antibodies and small-molecule gepants) represents a paradigm shift that has achieved remarkable clinical success in migraine prophylaxis [187,188]. However, as this strategy expands from neurological applications to a broader spectrum of CGRP-related pathologies, a core translational bottleneck emerges: the inherent nonspecificity of targeting such a ubiquitous physiological “conductor” [189]. Because CGRP exerts pleiotropic regulatory effects across vascular, neural, and immune networks, its systemic pharmacological blockade is conceptually akin to introducing a novel “corticosteroid-like” molecule. Shutting down this fundamental, multidimensional regulatory network inevitably triggers widespread “off-tissue” perturbations [190], necessitating a critical reevaluation of current clinical intervention paradigms [188,189].

### Real-world challenges: Mechanistic roots of nonspecificity and off-tissue adverse effects

This “corticosteroid-like” broad-spectrum inhibition provides a mechanistic rationale for the unique safety risks increasingly identified in real-world pharmacovigilance data [189]. Systemic antagonism indiscriminately abolishes the physiological protective functions of CGRP, translating basal neuroimmune mechanisms into tangible clinical liabilities:

First, elevated infection risks. By antagonizing CGRP’s basal regulation of mucosal immunity and immune cells (e.g., macrophages and T cells), systemic blockade indirectly compromises host immune surveillance. A recent meta-analysis corroborated this, demonstrating a potential increase in infectious adverse events (e.g., upper respiratory tract infections) among patients receiving preventive CGRP-targeting therapies [191].

Second, severe gastrointestinal complications. Given the widespread distribution of CALCRL/RAMP1 receptor complexes within the enteric nervous system, nonspecific blockade disrupts CGRP’s crucial regulation of gut motility and local reflexes. This directly explains why severe constipation has emerged as one of the most prominent adverse events in large-scale pharmacovigilance analyses of both gepants and anti-CGRP monoclonal antibodies [190,192].

Third, potential cerebrovascular risks. CGRP is an exceptionally potent endogenous microvasodilator. Under specific pathological stress (e.g., acute ischemia), this compensatory vasodilation is vital for maintaining tissue survival. Chronically depriving the body of this protective mechanism may expose or exacerbate cerebrovascular ischemic risks in susceptible populations-a limitation mirroring the real-world adverse event profiles of classical acute migraine medications, such as triptans [193].

### Uncharted territory: Biological costs of long-term blockade and “drug holiday” strategies

Beyond spatial nonspecificity, the temporal dimension of uncertainty poses an equally formidable challenge [189]. Because the widespread clinical application of anti-CGRP therapies is relatively recent, our understanding of the profound biological consequences of chronic or potentially lifelong deprivation of CGRP signaling remains fundamentally limited [193]. Sustained inhibition of this central neuroimmune hub raises a series of alarming mechanistic uncertainties, including compensatory receptor up-regulation, paradoxical hyperactivation of alternative proinflammatory pathways, and the potential waning of therapeutic efficacy over time [188,189]. Furthermore, the sophisticated neuroimmune orchestration of the CGRP axis extends to the pathogenesis of chronic pain disorders, most notably medication-overuse headache. Recent systemic evidence highlights that medication-overuse headache is characterized by a profound neuroimmune-epigenetic dysregulation [194]. Mechanistically, chronic exposure to acute migraine medications triggers a persistent elevation of systemic CGRP, which synergizes with immunological factors such as the P2X7R/NLRP3 inflammasome signaling in microglia to drive central sensitization. This process is further reinforced by epigenetic modifications, including aberrant DNA methylation and histone acetylation of genes regulating nociceptive plasticity and immune homeostasis. Such molecular remodeling shifts CGRP from a transient migraine mediator to a foundational driver of chronic cephalalgia, suggesting that targeting the neuroimmune interface—rather than simple neuropeptide blockade—is essential for mitigating the adaptive immunopathology associated with long-term pharmacological interventions in pain management [194]. Therefore, establishing evidence-based, standardized criteria for treatment discontinuation, dose-tapering protocols, or strategic “drug holidays” is an urgent clinical priority to balance sustained efficacy with the restoration of basal biological homeostasis [189]. Rigorous mechanistic investigations and continuous, long-term pharmacovigilance are indispensable to fully elucidate the long-term biological toll of perturbing this ubiquitous neuroimmune interface [191,192].

## Conclusion

**Fig. 8.**
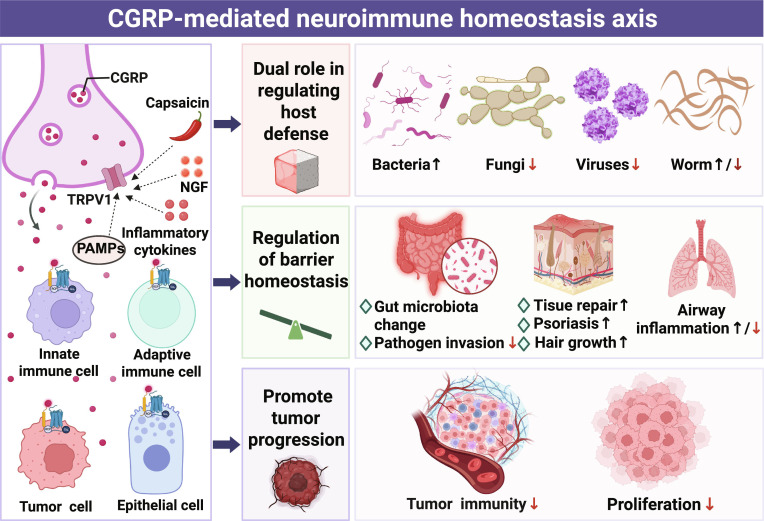
Calcitonin gene-related peptide (CGRP) neuroimmunological axis regulatory network*.* Schematic overview of the CGRP-mediated neuroimmune homeostasis axis and its context-dependent functional pleiotropy. CGRP is released from sensory neurons upon activation by transient receptor potential vanilloid 1 (TRPV1) agonists (e.g., capsaicin), nerve growth factor (NGF), pathogen-associated molecular patterns (PAMPs), or inflammatory cytokines, signaling through receptors on innate/adaptive immune cells, tumor cells, and epithelial cells. Host defense: Exhibits pathogen-specific dual functions—impairs bacterial clearance (↑), enhances antifungal (↓) and antiviral immunity (↓), and bidirectionally regulates antihelminth responses (↑/↓). Barrier homeostasis: Modulates gut microbiota composition, restricts pathogen invasion (↓), promotes tissue repair and hair growth (↑), exacerbates psoriasis (↑), and exerts bidirectional control over airway inflammation (↑/↓). Tumor progression: Facilitates tumorigenesis by suppressing antitumor immunity (↓) and promoting cancer cell proliferation (↑). This figure was created in BioRender. Gao, C. (2026) https://BioRender.com/wno9es9 (agreement number: IX29I91FHY)

The cumulative evidence synthesized herein establishes CGRP as a central hub in neuroimmune regulation, orchestrating a spectrum of biological outcomes that span host defense, tumor progression, and barrier homeostasis (Table 2 and Fig. 8). Moving beyond simplistic binary characterizations, our analysis reveals CGRP function as an actively regulated process shaped by 3 hierarchical contextual determinants: pathogen-associated molecular patterns, tissue-specific microenvironmental cues, and the dynamic interplay of cosignaling pathways. In bacterial infections, CGRP typically exerts immunosuppressive effects—impairing neutrophil recruitment, promoting M2 macrophage polarization, and inhibiting NLRP3 inflammasome activation—mechanisms exploited by pathogens to evade clearance. Conversely, in fungal or viral contexts, CGRP switches to a proinflammatory mode, activating IL-23/IL-17 axis signaling or reinforcing antiviral barriers, underscoring its remarkable functional plasticity.

**Table 2.** Summary table of CGRP’s roles, target cells, mechanisms, and therapeutic potential in various diseases

Specific diseases	Roles of CGRP	Target cells	Core mechanisms	Therapeutic potential
Bacterial infections (*S. aureus*, *Streptococcus*, *C. difficile*, *K. pneumoniae*, *P. aeruginosa*)	• Impairs host defense and exacerbates infection [42,46,48,57,63,68,72] • Alleviates inflammation in corneal infections [67]	Neutrophils [46,48,63], macrophages [48,67,68], monocytes [42], intestinal pericytes [57], meningeal macrophages [72]	• Inhibits neutrophil recruitment and bactericidal activity [46,48,63] • Promotes macrophage M2 polarization [48,67] • Suppresses NLRP3 inflammasome [68] • Induces IL-8 secretion by intestinal pericytes [57] • Activates PI3K/AKT pathway to drive macrophage M2 polarization [67]	• Apply CGRP receptor antagonists (BIBN4096) [74] • Inhibit CGRP release (BoNT/A) [64] • Locally block CGRP signaling to alleviate corneal inflammation [67]
Fungal infections (*C. albicans*, *A. fumigatus*, candidal osteomyelitis)	• Enhances antifungal defense [41,76] • Suppresses excessive inflammation [75,78,79]	DCs [41,76], macrophages [78,79], osteoclasts [75]	• Drives DCs to secrete IL-23, inducing IL-17 secretion by dermal γδ T cells [41,76] • Inhibits macrophage NLRP3 [78,79] • Induces Jdp2 to inhibit NF-κB, suppressing osteoclast activation [75]	• Target CGRP pathway to enhance antifungal immunity [41,76] • Utilize CGRP to inhibit inflammation in fungal osteomyelitis [75]
Parasitic infections (Helminths, *N. brasiliensis*)	• Restricts type 2 inflammation and inhibits parasite clearance [82,83]	ILC2 [82,83]	• Inhibits ILC2 proliferation and IL-13 secretion [82,83] • Activates CREB to regulate IL-5 [82]	Regulate CGRP levels to balance inflammation and parasite clearance [82,83] • Combine with NMU to improve antihelminth immunity [82]
Viral infections (HSV, HIV, SARS-CoV-2, influenza, LCMV)	• Blocks viral entry and enhances adaptive immunity [89,95,98,100,101]	Langerhans cells [89,95], B cells [100], T cells [101], airway epithelial cells [98]	• Down-regulates viral entry receptors (ACE2, 3-OS HS) [89,98] • Promotes germinal center reactions in B cells [100] • Activates CREB/ATF3 to induce T cell differentiation into Th1 cells [101]	• Apply CGRP analogs (SAX) to block viral entry [95] • Supplement CGRP to improve viral clearance [101,102]
Intestinal-related diseases (DSS-induced colitis, food allergy, CDI, *Salmonella* infection, TBI-related translocation)	• Maintains epithelial barrier and microbiota homeostasis [106,110,117,120,125] • Exacerbates CDI inflammation [57]	Goblet cells [125], ILC2 [120], Treg cells [106], intestinal pericytes [57], M cells [117]	• Promotes mucus secretion by goblet cells [125] • Inhibits KLRG1^+^ ILC2 activation [120] • Suppresses RORγ^+^ Treg proliferation [106] • Reduces M cell numbers and increases SFB levels [117] • Induces IL-8 secretion by intestinal pericytes [57]	• Activate CGRP–RAMP1 axis to repair intestinal barrier [125] • Target CGRP to improve colitis and food allergy [106,120] • Block CGRP signaling to alleviate CDI [57] • Induce CGRP secretion to reduce bacterial translocation [119]
Skin-related diseases (diabetic wounds, psoriasis, alopecia, atopic dermatitis)	• Promotes wound healing and hair growth [40,139] • Exacerbates psoriasis inflammation [144,149,150] • Suppresses AD inflammation [35]	T cells [35,149], neutrophils [40], macrophages [40], keratinocytes [150], DCs [144,149], fibroblasts [139]	• Inhibits IL-17 secretion by T cells [149] • Induces macrophages to release TSP-1 [40] • Drives DCs to secrete IL-23 [144,149] • Up-regulates Spp1 secretion by CD9^+^CD26^+^ fibroblasts [139] • Induces keratinocyte proliferation [150]	• Topically apply eCGRP to promote wound healing [40] • Use CGRP receptor antagonists (Rimegepant) to treat psoriasis [144] • Target CGRP pathway to balance inflammation in atopic dermatitis [35]
Pulmonary-related diseases (allergic airway inflammation, acute lung injury [ALI])	• Bidirectionally regulates airway inflammation [155,158,160] • Protects alveolar epithelium and alleviates ALI [162,164]	Th9 cells [155], DCs [158,163], ILC2 [160], alveolar epithelial cells [162], alveolar macrophages [163]	• Promotes IL-9 secretion by Th9 cells (proinflammatory) [155] • Inhibits DC maturation and ILC2 activation (anti-inflammatory) [158,160,163] • Activates AMPK/L-OPA1 pathway to protect mitochondria [162]	• Target RAMP1 to inhibit airway inflammation [160] • Utilize CGRP to improve ALI [162] • CGRP receptor antagonist (CGRP 8-37) confirms its protective effect [164]
Tumors (melanoma, HNSCC, OSCC, MTC, gastric cancer, PDAC)	• Promotes tumor growth and metastasis, inhibits antitumor immunity [36,171,173,175] • Aggravates cancer pain and progression in some cases [179,180,182,185]	CD4^+^ T cells [173], CD8^+^ T cells [36,171,173], DCs [175], tumor cells [180,182], CAFs [185], NK cells [185]	• Induces CD8^+^ T cell exhaustion (up-regulates PD-1/LAG3/TIM3) [36] • Inhibits DC maturation via cAMP-KLF2 pathway [175] • Activate Rap1-GTPase to induce tumor cell autophagy [180] • Inhibits CAFs secretion of IL-15 to weaken NK cell function [185] • Forms NGF–CGRP positive feedback loop [180,182,185]	• Combine CGRP receptor antagonists (Rimegepant, BIBN4096) with radiotherapy/immunotherapy [167,173] • Target CGRP–RAMP1 axis to block tumor proliferation [36,182] • Block NGF–CGRP positive feedback to suppress tumor progression and analgesia [185]
Autoimmune/inflammatory diseases (SJS/TEN, atopic dermatitis, psoriasis, allergic asthma)	• Enhances CD8^+^ T cell cytotoxicity and exacerbates tissue damage (SJS/TEN) [186] • Suppresses type 2 inflammation (atopic dermatitis) [35] • Exacerbates psoriasis inflammation [144,149,150] • Bidirectionally regulates airway inflammation (allergic asthma) [155,158,160]	CD4^+^ T cells [35], CD8^+^ T cells [186], DCs [144,158], ILC2 [35,120,158] keratinocytes [150], γδ T cells [149]	• Activates HCN2 channel on CD8^+^ T cells (SJS/TEN) [186] • Inhibits IL-31 and type 2 inflammation (atopic dermatitis) [35] • Drives DCs to secrete IL-23 and induces keratinocyte proliferation (psoriasis) [144,149,150] • Promotes Th9 secretion of IL-9 and inhibits DC/ILC2 activation (allergic asthma) [155,158,160]	• Block CGRP–RAMP1 signaling (SJS/TEN) [186] • Target CGRP pathway to balance inflammation (atopic dermatitis) [35] • Use CGRP receptor antagonists to treat psoriasis [144] • Precisely regulate inflammation with CGRP receptor antagonists (allergic asthma) [160]

CGRP, calcitonin gene-related peptide; NGF, nerve growth factor; ILC2, group 2 innate lymphoid cell; DC, dendritic cell; RAMP1, receptor activity-modifying protein 1; cAMP, cyclic adenosine monophosphate; NF-κB; nuclear factor kappa B; NK, natural killer; BoNT/A, botulinum neurotoxin A; IL-8, interleukin-8; PI3K, phosphoinositide 3-kinase; NLRP3, NLR family pyrin domain containing 3; Jdp2, Jun dimerization protein 2; CREB, cAMP response element-binding protein; 3-OS HS, 3-O-sulfated heparan sulfate; ACE2, angiotensin-converting enzyme 2; ATF3, activating transcription factor 3; Th1, type 1 helper; SFB, segmented filamentous bacteria: TBI, traumatic brain injury; KLRG1, killer cell lectin-like receptor G1. DSS, dextran sulfate sodium; eCGRP, engineered CGRP; TSP-1, thrombospondin-1; Spp1, secreted phosphoprotein 1; AMPK, AMP-activated protein kinase pathway; L-OP1, long form of optic atrophy 1; PDAC, pancreatic ductal adenocarcinoma; OSCC, oral squamous cell carcinoma; KLF2, Krüppel-like factor 2; GTPase, guanosine triphosphatase; CAFs, cancer-associated fibroblasts; SJS, Stevens–Johnson syndrome; TEN, toxic epidermal necrolysis; HCN2, hyperpolarization-activated cyclic nucleotide-gated channel 2; NMU, neuromedin U

Within the TME, CGRP emerges as a critical mediator of immune evasion. It directly induces CD8^+^ T cell exhaustion through checkpoint receptor up-regulation, disrupts DC antigen presentation via cAMP-KLF2 signaling, and confers cytoprotective autophagy to cancer cells. These conserved mechanisms, observed across melanoma, medullary thyroid carcinoma, and OSCC, position CGRP as a promising therapeutic target for cancer immunotherapy.

At barrier interfaces—gastrointestinal, cutaneous, and respiratory—CGRP functions as a homeostatic rheostat. It modulates goblet cell mucus secretion, shapes microbiota composition, and calibrates inflammatory responses, with its dysregulation contributing to pathologies like psoriasis and allergic asthma. Notably, recent studies highlight CGRP’s role in maintaining intestinal barrier integrity through RORγ^+^ Treg modulation, a finding with direct implications for inflammatory bowel disease.

The mechanistic complexity of CGRP signaling presents fertile ground for future inquiry. Three overarching questions demand attention: (a) Can we decode the “CGRP receptor code”—combinatorial expression of CALCRL-RAMP complexes and signaling adaptors that dictates functional outcome? Single-cell sequencing studies already suggest cell-type-specific adaptor expression (e.g., FADD in tumor T cells versus stromal interaction molecule in SJS/TEN T cells) as a key determinant. (b) How is the “neuroimmune connectome” organized? Emerging data on sensory neuron-immune cell synapses, particularly in barrier tissues, call for spatial transcriptomic mapping to identify circuit-specific regulatory motifs. (c) To what extent does temporal dynamics—CGRP release kinetics and duration—influence its paradoxical effects? Optogenetic models permitting precise temporal control of CGRP secretion will be invaluable here.

Translating these insights requires integrating cutting-edge technologies into a cohesive research framework:

1. Multiomics mapping: Single-cell and spatial transcriptomics, coupled with proteomics and metabolomics, to define tissue-specific “signal codes”—for instance, how microbiota-derived short-chain fatty acids regulate RAMP subtype expression.
2. Organoid-neuron coculture systems: Development of intestinal, cutaneous, and pulmonary organoids integrated with dorsal root ganglion neurons to model neuroimmune crosstalk ex vivo, enabling mechanistic dissection of CGRP’s temporal effects.
3. Structure-guided drug design: Leveraging cryo-EM structures of CGRP-RAMP complexes to develop isoform-specific modulators, such as b-CGRP agonists for barrier repair or CALCRL–RAMP1 antagonists for cancer immunotherapy.
4. Microbiota-neuroimmune axis targeting: Identification of microbial taxa (e.g., *Muribaculaceae*) that modulate CGRP secretion, opening avenues for probiotic-based adjuvant therapies.
5. Precision clinical translation: Prospective trials stratifying patients by CGRP isoform expression, RAMP subtype, and microenvironmental markers, paired with tissue-specific delivery systems (e.g., inhalable formulations for asthma and topical hydrogels for psoriasis).
6. Unraveling sex dimorphism: Striking sex disparities exist in CGRP-driven pathologies (e.g., migraine and autoimmune disorders). Systematically delineating how gonadal hormones intersect with CGRP receptor signaling and adaptor recruitment will be essential for developing sex-tailored immunotherapies.

Ultimately, advancing CGRP research requires a paradigm shift from descriptive observation to mechanistic engineering. By deciphering its signaling circuitry, developing targeted modulators, and implementing precision delivery strategies, we can transform CGRP from a pleiotropic mediator into a rationally tunable therapeutic target—unlocking its potential to treat infectious diseases, cancer, and inflammatory disorders [195–198].
